# Developmental and Transcriptomic Responses in Sea Urchin Larvae to an Urban‐Associated Pollutant

**DOI:** 10.1002/ece3.72183

**Published:** 2025-09-17

**Authors:** Madison L. Armstrong, Sindhu Bala, Rachael A. Bay

**Affiliations:** ^1^ Center for Population Biology University of California Davis CA USA; ^2^ Te Whare Wānanga o Waitaha University of Canterbury Christchurch New Zealand

**Keywords:** larval development, pollution, *Strongylocentrotus purpuratus*, transcriptomics, urbanization

## Abstract

Urban environments provide a unique opportunity to investigate the impacts of novel stressors on organismal performance. Marine intertidal zones exist at the transition from sea to land, where they are exposed to a unique suite of stressors, including those associated with wastewater outflow, sewage effluent, and coastline development. Although studies have shown that compounds found in wastewater, including endocrine disrupting chemicals (EDCs), can affect the survival and development of marine organisms, the mechanisms for those effects are relatively unknown. Our study investigates the developmental and transcriptomic responses to a common EDC, nonylphenol, using the Pacific purple sea urchin ( *Strongylocentrotus purpuratus* ) as a model system. Beginning exposure prior to fertilization, we found that nonylphenol impacts only materialize 24 h postfertilization when the embryonic transcriptome begins to be expressed, and these impacts vary significantly by mate pair. In addition, survival was lowest at the lowest concentration of nonylphenol. Transcriptomic patterns also varied by chemical concentration and developmental stage, with ribosomal genes differentially expressed among different treatments at both early and later larval stages. We also found a strong parental effect: survival, morphology, developmental abnormalities, and gene expression vary among mate pairs despite all of the adult urchins coming from the same population. This potentially suggests standing within‐population variation, which may impact evolutionary responses to anthropogenic stress. Overall, our study finds that nonylphenol affects survival, morphology, and gene expression at early life history stages, and that more work needs to be done to understand intraspecific variation in those effects.

## Graphical abstract

Marine intertidal organisms exist at the transition from sea to land, where they are exposed to a unique suite of stressors including those associated with wastewater outflow, sewage effluent, and coastline development. Exposing sea urchin larvae to several concentrations of a common urban‐associated chemical, we identified impacts on survival, morphology, and gene expression. We also observed inconsistent patterns of developmental abnormalities between mate pairs, suggesting higher within‐population variation than expected in response to marine urban environments.

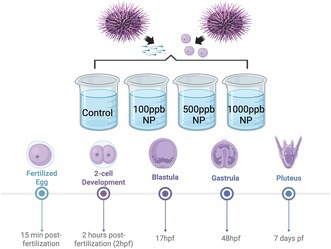

## 1 Introduction

Urban environments represent a complex suite of habitat characteristics. Although each urban environment is different, common environmental shifts include habitat fragmentation, increases in temperature, light, and noise, and the introduction of novel stressors (i.e., pollutants). In recent years, it has become increasingly apparent that urban environments can have strong and predictable effects on organisms (Alberti et al. 2020; Des Roches et al. 2021; Diamond et al. 2018; Johnson and Munshi‐South 2017; Lambert et al. 2021). Previous studies have shown that the combination of stressors in urban environments can alter phenotypic, physiological, and genomic variation (Campbell‐Staton et al. 2020; Diamond et al. 2018; Yilmaz et al. 2021). For example, physiological stress responses to handling in European blackbirds were lower in urban populations than in nonurban populations, suggesting that urban populations may be already acclimated or adapted to a stressful environment (Partecke et al. 2006). In another example, water pollution disrupts reproductive barriers in swordtail fish species, resulting in increased hybridization (Moran et al. 2025; Ramirez‐Duarte et al. 2025). With few exceptions (Park et al. 2025; Puritz and Toonen 2011; Whitehead et al. 2017), the vast majority of urban ecology and evolution studies have involved terrestrial systems; we know little about how the plethora of anthropogenic stressors on urban coastlines impacts marine organisms (Alter et al. 2021).

The marine intertidal zone lies at the interface between sea and land, so intertidal organisms face a complex mixture of urban stressors, including stormwater runoff, sewage wastewater, and coastline development. One particular source of novel stress is through chemicals originating in household and industrial products, including endocrine disrupting chemicals (EDCs), which are present in higher concentrations in urban coastlines (Björklund et al. 2009; Bressy et al. 2011; David et al. 2009; Dodder et al. 2014; Maruya et al. 2014). EDCs have also been shown to have negative impacts on a range of species (Vos et al. 2000), including developmental abnormalities in sea urchins (Arslan and Parlak 2007; Roepke et al. 2005) and mussels (Balbi et al. 2016; Lavado et al. 2006). Such localized stressors could impact survival, reproduction, and dispersal, ultimately cascading to population‐level effects (Larsson et al. 2016; Laufer et al. 2013; Liu et al. 2011; Puritz and Toonen 2011). For example, Laufer et al. (2013) suggest that a particular class of EDCs, alkylphenols, affects survival and molting in lobsters and contributed to a major die‐off event in Long Island Sound. Developing a mechanistic understanding of how chemicals on urban coastlines impact early developmental stages is important for understanding the long‐term effects of pollution on the persistence of marine populations.

Transcriptomics data can provide a deeper understanding of the mechanisms involved in stress response, especially paired with survival, developmental, and/or behavioral data (Gleason 2019). For example, Balbi et al. (2016) identified patterns of differential expression contributing to developmental abnormalities in mussel larvae exposed to Bisphenol A. Gene expression data can provide insight into which genes are differentially regulated after exposure to a particular stressor and also allow for comparison of different responses to stress across developmental stages. In the sea urchin *Heliocidaris erythrogramma*, exposure to ocean acidification stress elicits a distinct gene expression response at different developmental stages, with gene identity, magnitude of response, and variance in response varying between embryo, larval, and juvenile stages (Devens et al. 2020). However, few studies pair transcriptomic and developmental data to provide a holistic understanding of exposure pathways in marine organisms.

In this study, we examined the effects of different levels of EDC exposure in Pacific purple sea urchin ( *Strongylocentrotus purpuratus* ) larvae. *S. purpuratus* is a widespread marine invertebrate species, ranging from Alaska to Baja California. They are a valuable model for understanding physiological and developmental pathways in other echinoderms and marine invertebrates with biphasic life cycles (planktonic larvae and benthic adult). *S. purpuratus* are broadcast spawners, with each female producing millions of eggs per spawning, and spawning is easily achievable in the lab (Adams et al. 2019). The eggs themselves are relatively large and transparent, making microscopic visualization easier than it is for other urchin species (Adams et al. 2019). The developmental rate of *S. purpuratus* larvae is well recorded, and they have a short generation time, reaching the swimming and feeding larval stage (pluteus) in several days. This species is.

A common developmental model used in both ecotoxicology and evo‐eco studies, and previous work has established developmental timelines and abnormality classifications that can be used in this current study (Cunningham et al. 2020; Roepke et al. 2005; Shore et al. 2022; Uibel 2016).

We investigate responses to three concentrations of an EDC common in urban waterways, nonylphenol. Nonylphenol is a subclass of alkylphenols that makes up 80% of alkylphenols in sewage runoff (David et al. 2009). Alkylphenols are highly water‐soluble and are used in a variety of industrial and household products, including pesticides, herbicides, pharmaceuticals, and plastics. EDCs actively alter the efficacy of hormones, particularly thyroid and steroid hormones (Darbre 2022). The Mussel Watch program, examining 87 intertidal sites for the concentration of various EDCs, found evidence of alkylphenols at all of their sites, with increasing concentrations depending on proximity to urbanization and runoff (Dodder et al. 2014; Maruya et al. 2014). We combine measures of survival, development, and gene expression to understand the effects of nonylphenol on early developmental stages. Specifically, we ask: (i) how different concentrations of nonylphenol affect survival, development, and gene expression, (ii) how the response to nonylphenol exposure differs across developmental stages, and (iii) which genes and biological pathways respond to nonylphenol exposure.

## 2 Methods

### 2.1 Collections

We obtained adult individuals from an urchin population originally collected from Stillwater Cove (38.539677, −123.290377) and maintained at the University of California, Davis Bodega Marine Laboratory by the Aquatic Resource Group. Individuals were housed in a common garden environment within a flow‐through seawater system to mimic standard Bodega Bay temperatures (~13.8°C for July/August water temperatures) for several months and fed kelp (*Macrocystis pyrifera*) ad libitum to ensure good reproductive condition before spawning was induced (Strathmann 1987). We collected 10 adults from this larger lab population to ensure a high likelihood of obtaining at least four males and four females for the full experiment.

### 2.2 Spawning, Fertilization and Culture Maintenance

To induce spawning, we injected 2 mL of 0.5 M KCl into the peristomal membrane tissue surrounding Aristotle's lantern (mouth) (Adams et al. 2019). If the individual was female, it was placed in a beaker with filtered sea water (FSW) so that eggs could be collected at the bottom, and if the individual was male, it was placed oral side up in a small beaker to condense and keep sperm inactive until fertilization. Sperm was kept on ice to increase longevity and decrease sperm motility before fertilization was conducted. Once all individuals were successfully spawned, each group of eggs was washed three times in FSW. After the final wash, we measured density to ensure each beaker was at approximately 10–20 eggs/mL to decrease the risk of overcrowding (Hodin et al. 2019). A small portion of the female's eggs was then split into four beakers, representing the control and three nonylphenol exposures in the study. Beakers were then filled completely with their corresponding treatment water: (1) Control, (2) 100 ppb nonylphenol, (3) 500 ppb nonylphenol, and (4) 1000 ppb nonylphenol.

We then added concentrated sperm to each beaker that contained eggs, matching only one male to each female to establish four individual mate pairs, or families, with 16 beakers total. We refer to these families as mate pairs 1–4 throughout the rest of this paper. We set up each 1000 mL beaker with a stir paddle, following the previous methods established by Strathmann (1987) to keep larvae evenly distributed, maintain water oxygenation, and ensure optimal larval growth (Hodin et al. 2019). Stirring was initiated after developmental success counts to avoid damaging the embryos. Sperm was washed out after fertilization success was confirmed, 15 min after sperm and eggs were mixed. We cleaned the beakers with a reverse filtration method detailed in Hodin et al. (2019) and refilled them with the appropriate control or nonylphenol treatment water on day 3 and day 6 of the experiment. We maintained larval cultures at 15°C and at 35 ppm salt in FSW.

### 2.3 Chemical Treatments

**FIGURE 1:**
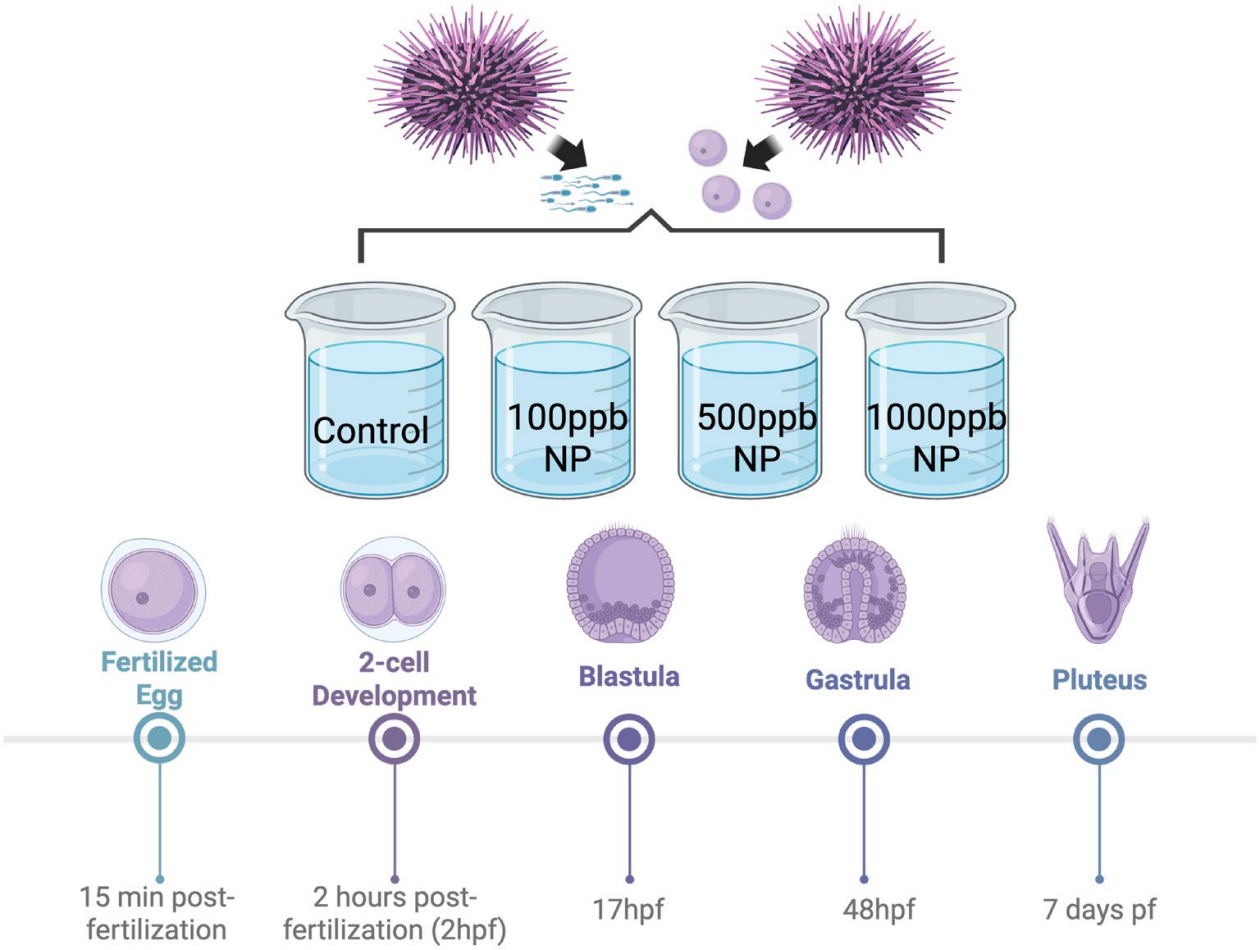
Schematic for experimental design for each mate pair in this study. Males and females were spawned, and gametes were combined and placed into the four treatments: Control, 100 ppb nonylphenol (NP), 500 ppb NP, and 1000 ppb NP. Data was then collected at the stages highlighted above, from fertilization success at 15 min postfertilization to the pluteus stage at 7 days postfertilization. Treatments were held constant throughout the study. Figure created in BioRender. Armstrong, M. (2025) https://BioRender.com/l5gs0wc.

Nonylphenol is highly prevalent in sewage runoff (David et al. 2009), and previous research has shown that nonylphenol resulted in extremely high proportions of developmental abnormalities, with 73% of oyster larvae exposed to 100 μg/L (approximately 100 ppb for nonylphenol) developing abnormally (Nice et al. 2000). Preliminary experiments in a different urchin species, the painted urchin ( *Lytechinus pictus* ), found no effect of nonylphenol at concentrations lower than 100 ppb, so we chose to test only concentrations 100 ppb and higher (Bala, Hawthorne, Armstrong, and Bay, unpublished). Given previous research, the treatments we chose for the four treatment levels were (1) Control, (2) 100 ppb nonylphenol, (3) 500 ppb nonylphenol, and (4) 1000 ppb nonylphenol. Dimethyl sulfoxide (DMSO) was added to our nonylphenol concentrations at 0.04 mL DMSO per 1 μL nonylphenol to ensure that nonylphenol was not stuck to the edges of the glassware and the density of nonylphenol was also accounted for (0.937 g/mL). No effect of DMSO alone has been observed in previous studies, so we did not include a DMSO‐only control (Shore et al. 2022). The lowest concentration was chosen to mirror the Nice et al. (2000) paper, with 500 and 1000 ppb similar to the research by Björklund et al. (2009) and Bressy et al. (2011). Seawater used for solutions was ambient Bodega Bay seawater (pH ~ 8.1), and the experiment was conducted in an unheated room used for experiments with local invertebrates (average temperature 13.8°C) at the Bodega Marine Laboratory. Exposure began when the unfertilized eggs and sperm were combined and was held constant throughout the study by cleaning and refilling beakers every few days with standardized solutions stored in carboys. This ensured that larvae were exposed to nonylphenol during fertilization and throughout the development stage. The full experimental design is shown in Figure 1.

### 2.4 Fertilization and Development Success

We quantified fertilization and development success using the same methods presented in Shore et al. (2022). To quantify fertilization success, we mixed the beakers to ensure an even sampling of eggs and collected six 500 μL water samples from each beaker (16 beakers total, 4 families × 4 treatments). We then counted the number of fertilized and unfertilized eggs present. We then assessed the average proportion of fertilized eggs by calculating the number of fertilized eggs over the total number of eggs. Development success was calculated in a similar way: we collected six water samples from each beaker 2 h postfertilization (hpf)—when the embryos should be at the 2‐cell stage—and counted the proportion of developed embryos divided by the total embryos observed to get the average development success per beaker. For each beaker, we averaged both fertilization and development proportions across the six replicate samples.

### 2.5 Larval Survival Assessment

To assess how different concentrations of nonylphenol may impact survival, we compared culture density changes between two developmental stages: blastula (17 hpf) and pluteus (7 days‐pf). We sampled 10 replicate samples of 500 μL each per beaker and counted the number of living individuals in each. Then, for each individual beaker, we averaged the number of embryos across the 10 replicates at the blastula stage and did the same for the pluteus stage. We then calculated the survival between the stages by subtracting the average number of pluteus from the average number of blastula, then dividing by the average number of blastula. We used this value to test whether survival between the two stages varied with different concentrations of nonylphenol treatment across the four mate pairs. To account for between‐beaker density variation, we compared survival between these two stages rather than comparing densities across beakers at a single timepoint.

### 2.6 Larval Morphology and Developmental Abnormalities

To assess whether morphology and developmental abnormalities were impacted by different concentrations of nonylphenol, we collected one 10 mL sample from each beaker at three developmental stages (blastula, gastrula (48 hpf), and pluteus) and preserved them with 0.1% glutaraldehyde. These preserved samples were then imaged using raised coverslips under a Nikon Eclipse E600 phase microscope at 10× magnification. After collecting image data for all 16 beakers, we assessed developmental abnormalities using previously reported abnormality types in *S. purpuratus*. These abnormality types included packed blastulae, gastrulae with degraded stomachs, undeveloped gastrulae, and a multitude of abnormalities in the pluteus stage, including abnormal arms, degraded stomach, abnormal skeleton, arrested development, and bell shape (Cunningham et al. 2020; Pillai et al. 2003; Roepke et al. 2005; Torres‐Duarte et al. 2016, 2017; Uibel 2016). After presence/absence classification of abnormalities for each imaged larva at each developmental stage, we then assessed the proportion of larvae that were abnormal in each beaker.

We used Fiji (GPLv3+, v. 2.16.0, built on top of ImageJ2) to take stage‐specific measurements on the remaining normal larvae that were preserved. All measurements taken across the three stages are shown in more detail in Figure S1. For the blastula stage, the diameter was taken to assess overall embryo size (2A). For the gastrula stage, height (2B) and stomach length (2C) were measured to assess the progression of development. For the pluteus stage, we collected three measurements—body length (2D), arm length (2E), and stomach area (2F) that have been previously established as valuable morphological measurements when understanding the impacts of environmental stress on pluteus development (Pespeni et al. 2013; Shore et al. 2022; Wong and Hofmann 2020).

### 2.7 Statistical Analysis

All statistics were performed in RStudio 4.3.1. For analyses of fertilization success, development success, survival, and abnormality proportion, we used linear models to fit the data, including mate pair and treatment as fixed effects. Although we intended for mate pairs to be biological replicates, which would be included as a random effect, it became obvious that variation among mate pairs was an interesting and important source of variation that we should estimate, so we included mate pairs as a fixed effect. However, we did not have multiple beakers for within mate pair–treatment combinations, so we can't account for an interactive effect. We then used ANOVAs to test for the significance of the mate pair and treatment level.

Although we used ANOVA tests for overall differences among treatment levels, for survival, we wanted to explicitly ask whether each treatment was lower than the control while also accounting for the mate pair. We calculated a relative survival value for each nonylphenol treatment by subtracting survival in each treatment from that survival for that mate pair in the control treatment. We then used one‐tailed *t*‐tests to determine whether each treatment had significantly lower survival. We used a Bonferroni correction to account for multiple testing.

For morphological measurements, we fit the data to linear models with two predictor variables, mate pair and treatment. In this case, since the unit of replication was a single larva (i.e., we have individual measurements), we also included an interaction term. Significance of factors was estimated using ANOVA. All mean and standard deviation information was gathered for plotting using the dplyr package (v. 1.1.4).

### 2.8 Transcriptomics

We collected samples at the gastrula and pluteus stages for transcriptomic analyses to investigate how larval development and gene expression patterns are affected by treatment and mate pair. Approximately 900 larvae were collected from each beaker, to represent each mate pair, treatment, and stage, and preserved in Trizol. Although we also attempted RNA extraction from blastula samples, we were unable to extract a sufficient quantity of RNA from those, likely because of the small size of the blastula. RNA was extracted from each pooled sample following a Zymo Directzol Kit protocol. Some of our beakers had low survival (especially those belonging to mate pair 2), and we did not have enough larvae for RNA‐Seq. We ended up with a total of 26 RNA samples across the two stages: 13 gastrula RNA samples (missing MP2 control, and MP2 1000 ppb and MP3 500 ppb) and 13 pluteus RNA samples (missing MP4 100 ppb, and MP2 500 ppb and 1000 ppb). We sent total RNA to the UC Davis Genome Center, where they prepared 3′ Tag‐Seq libraries, with each experimental beaker individually barcoded, then all samples were combined into a lane of sequencing using Element Biosciences AVITI Sequencing. The average number of reads per sample was 8.6M with a range of 6.7M‐11M, and we used multiqc (v.1.23) to visualize sample quality (Ewels et al. 2016). An RNASeq snakemake pipeline designed by J. Griffiths (https://github.com/JoannaGriffiths/RNASeq‐snakemake‐pipeline) was modified and used to run FastQC v. 0.11.9 (https://www.bioinformatics.babraham.ac.uk/projects/fastqc/), FastP v. 0.23.4 (Chen 2023), and Salmon v. 1.10.1 (Patro et al. 2017) with default parameters. We used the 2019 *Strongylocentrotus purpuratus* reference genome and reference transcriptome (GCF_000002235.5) from NCBI RefSeq (BioSample ID SAMN00829422, Baylor College of Medicine). This pipeline performs adapter and quality trimming and uses the quasi‐mapping approach in Salmon to generate a matrix with the estimated abundance for each transcript in each sample. A pre‐filtering step was done to remove genes with counts less than or equal to 10 across all samples to reduce low‐abundance transcripts. Analyzing gastrula and pluteus samples separately, we used the R package DESeq2 (10.18129/B9.bioc.DESeq2 (Love et al. 2014)). Data were normalized using a parametric fit for the dispersion of the data via a variance stabilizing transformation, normalizing by library size.

We conducted a principal component analysis (PCA) using variance‐stabilized transformed values and the top 500 features by variance. We used linear models to test whether sample loadings on PC axes could be explained by mate pair or nonylphenol treatment with lm(PC1 ~ Matepair + Treatment). We then used a likelihood ratio test (LRT) through DeSeq2 (Love et al. 2014) for data within each development stage (gastrula and pluteus) to identify genes that vary among treatment levels. For each gene, the LRT compares a model that includes both mate pair and treatment (expression ~ mate pair + treatment) to a null model with only mate pair (expression ~ mate pair). Significant genes vary among treatments after accounting for the effects of the mate pair. The LRT is useful because it allows us to compare all treatments simultaneously rather than conducting pairwise tests among treatments. Finally, we used the Ensembl bioMart database (Smedley et al. 2009) to extract annotations and Gene Ontology (GO) terms for differentially expressed genes. We used topGO (v. 2.56.0) (Alexa and Rahnenfuhrer 2017, 2009) to identify GO term enrichment using a Fisher's test with all expressed genes as the background.

## 3 Results

### 3.1 Fertilization and Development

Fertilization and development success were consistently high across all mate pairs and treatments. We saw no significant difference in fertilization success between treatments (two‐way ANOVA, *F*_3,3_ = 1.065, *p* = 0.411) or across mate pairs (two‐way ANOVA, *F*_3,3_ = 1.835, *p* = 0.021). A similar pattern was seen for development success, with no significant differences between treatments (two‐way ANOVA, *F*_3,3_ = 1.644, *p* = 0.247) or across mate pairs (two‐way ANOVA, *F*_3,3_ = 2.333, *p* = 0.142) (Figure S2). Between 96.4% and 100% of the eggs were fertilized for all four treatments across all beakers (*n* = 16). Development success ranged from 90% to 100% for all beakers except for the control beaker for mate pair 2, which had 75% development success. However, since this was only seen in the control of one mate pair, this decrease in development success was not due to nonylphenol treatment.

### 3.2 Larval Survival

**FIGURE 2:**
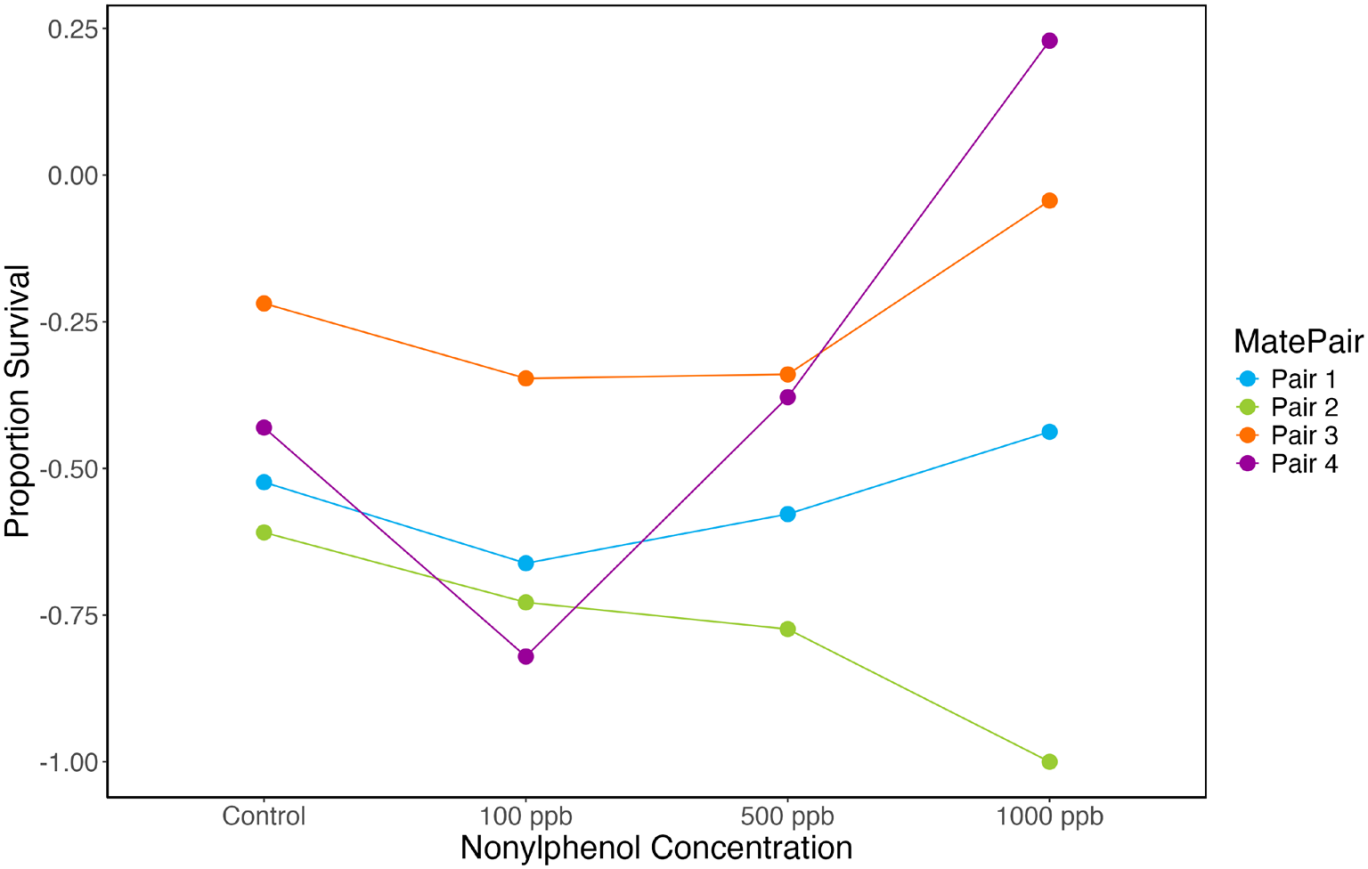
Proportion survival between blastula and 7 dpf pluteus across treatments. The *X*‐axis represents the four treatment conditions, and the *Y*‐axis shows the proportion of survival from the blastula to the 7‐day pf. Different colors highlight different mate pairs and how their survival changes across nonylphenol concentrations. The general trend shows slightly lowered survival in the 100 ppb nonylphenol treatment with more variable responses at higher concentrations of nonylphenol; however, this is not statistically significant (one‐sample *t*‐test, df = 3, *p* = 0.060). Survival significantly differed across mate pairs (two‐way ANOVA, *F*_3,3_ = 4.004, *p* = 0.046).

Mate pairs had different rates of survival (Figure 2; two‐way ANOVA, *F*_3,3_ = 4.004, *p* = 0.046). Densities of larvae per mL ranged from 9 to 40 in the blastula stage and 0 to 15 in the pluteus stage. Although overall survival did not vary across treatments (two‐way ANOVA, *F*_3,3_ = 1.320, *p* = 0.327), we did find that when testing relative survival values of each nonylphenol treatment against control, survival in the 100 ppb nonylphenol treatment was slightly lower, though significance was marginal after correction for multiple tests (one‐sample *t*‐test, df = 3, Bonferroni‐corrected *p* = 0.060).

### 3.3 Developmental Abnormalities and Morphology

**FIGURE 3:**
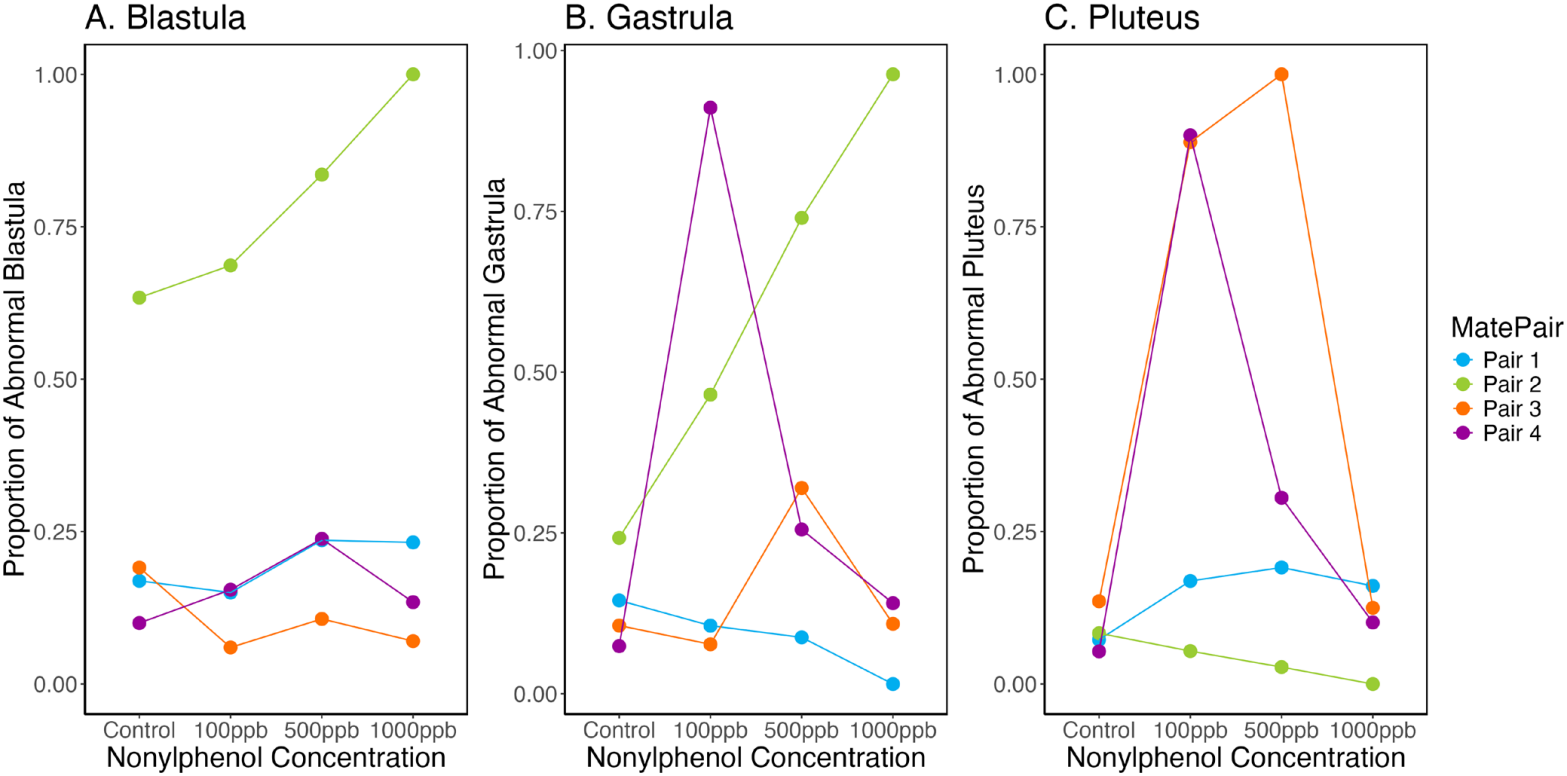
Proportion of abnormalities across all three developmental stages: Blastula (Left), Gastrula (Center), and Pluteus (Right). Different mate pairs appear to spike in abnormalities at different developmental stages, with mate pair 2 (green) showing a high proportion of abnormalities at the blastula stage onward (lower in pluteus because of decreased survival) and mate pair 3 (orange) and 4 (purple) showing higher abnormalities starting in the gastrula and pluteus stages, respectively. Mate pair 1 (blue), on the other hand, shows no spikes in abnormalities and rather stays at a low proportion throughout the experiment. Across all three stages, there was no effect of treatment observed (two‐way ANOVA, blastula: *P* = 0.3505; gastrula: *P* = 0.595; pluteus: *P* = 0.121), but there was an effect of mate pair in blastula stage (two‐way ANOVA, *F*_3,3_ = 48.240, *p* < 0.0001) and a slight trend in the gastrula stage (two‐way ANOVA, *F*_3,3_ = 2.975, *p* = 0.089).

Across all mate pairs, treatment, and developmental stage combinations, we examined 20–106 blastula per beaker (average = 81 per beaker), 27–86 gastrula per beaker (average = 61 per beaker), and 20–110 pluteus larvae per beaker (average = 66 per beaker) for abnormalities. Proportions of abnormal larvae varied dramatically, from 0% to 100%. The proportion of abnormalities ranged drastically within each developmental stage: from 6% to 100% for blastula, 2% to 96% for gastrula, and 0% to 100% for pluteus. Developmental abnormalities varied significantly across mate pairs for the blastula stage (two‐way ANOVA, *F*_3,3_ = 48.240, *p* < 0.0001). There was a marginally significant difference in developmental abnormalities between mate pairs for the gastrula stage (two‐way ANOVA, *F*_3,3_ = 2.975, *p* = 0.089), but no significant difference in the pluteus stage (two‐way ANOVA, *F*_3,3_ = 2.806, *p* = 0.101). Across all three stages, there was no effect of nonylphenol treatment observed (two‐way ANOVA, blastula: *p* = 0.351; gastrula: *p* = 0.595; pluteus: *p* = 0.121). Though we cannot statistically test the interaction between mate pair and treatment, there appear to be highly inconsistent patterns across mate pairs relative to treatment (Figure 3). Mate pair 2 shows a high proportion of abnormalities starting in the blastula stage and continuing in the gastrula stage. This appears higher in the higher nonylphenol treatments. However, mate pair 2 has very few abnormal pluteus, which, paired with very low survival in this family, might suggest that individuals abnormal at the earliest developmental stages did not survive to pluteus. Mate pair 4 shows a high proportion of abnormalities starting at the gastrula stage, and mate pair 3 shows a high proportion of abnormalities starting only at the pluteus stage. On the other hand, mate pair 1 showed a low proportion of abnormalities throughout the experiment.

There were mate pairs, treatment, and interactive effects on the measured morphological traits across the three developmental stages, but the effects of treatment were inconsistent across mate pairs and stages (Statistics and visualization of this data shown in Figures S3–S5). For example, at the blastula stage, mate pair 4 showed smaller blastulae with increasing nonylphenol concentration, whereas mate pair 3 showed the opposite trend with larger blastulae at higher concentrations of nonylphenol. The other morphological measurements taken had similarly inconsistent trends, with gastrula height and pluteus body length varying across different concentrations with no obvious shared response to our treatment concentrations. The significance of nonylphenol treatment in these comparisons may be driven by the extremely large number of larvae measured per beaker (blastula: mean = 56 embryos, range = 0–93; gastrula: mean = 33 embryos, range = 1–73; pluteus: mean = 18 larvae, range = 0–56). The statistics for all morphological measurements are reported in Table S1.

### 3.4 Gene Expression

**FIGURE 4:**
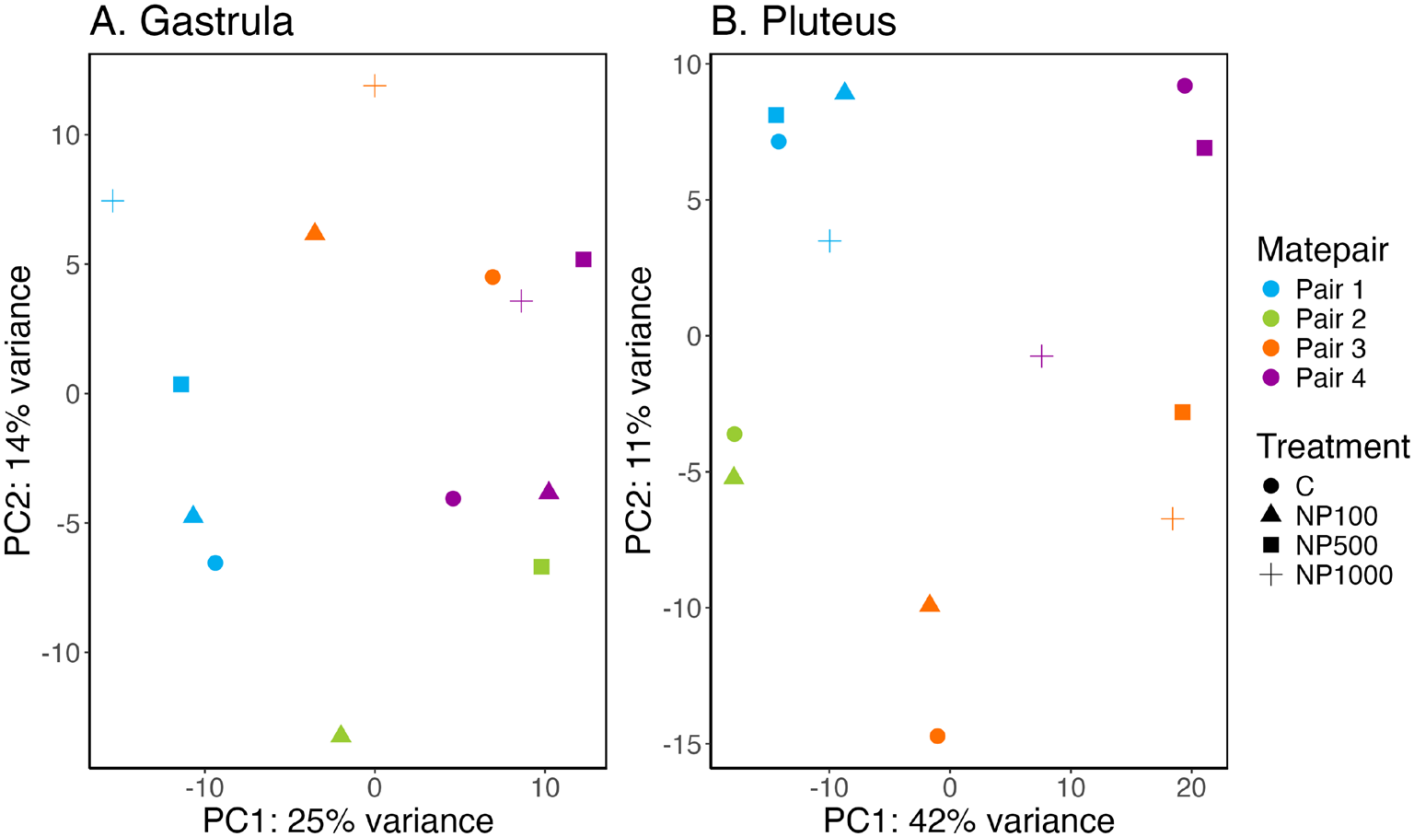
PCAs showing gene expression samples grouping by mate pair (colors) and treatment (shapes) across two developmental stages, gastrula (A) and pluteus (B). (4A): For the gastrula stage, PC1 was highly driven by mate pair (25% variance, two‐way ANOVA, *F*_3,3_ = 15.882, *p* = 0.003). Meanwhile, PC2 was driven by mate pair (14% variance, two‐way ANOVA, *F*_3,3_ = 27.844, *p* < 0.001) and treatment (two‐way ANOVA, *F*_3,3_ = 15.997, *p* = 0.003). (4B): For the pluteus stage, only the mate pair significantly drives the variation seen in both PC1 (34% variance, two‐way ANOVA, *F*_3,3_ = 10.621, *p* = 0.008) and PC2 (13% variance, two‐way ANOVA, *F*_3,3_ = 11.487, *p* = 0.007).

Overall gene expression patterns were driven by mate pair in both developmental stages, with additional separation by treatment in the gastrula but not pluteus stage (Figure 4). We obtained an average of 8.6 million reads (range 6.69–10.95 million reads) per pooled larval sample. PCA of gastrula samples shows mate pairs separating along both PC1 (25% variance, two‐way ANOVA, *F*_3,3_ = 15.882, *p* = 0.003) and PC2 (14% variance, two‐way ANOVA, *F*_3,3_ = 27.844, *p* < 0.001). However, PC2 also separated different treatments, with lower concentrations falling at lower PC2 scores (two‐way ANOVA, *F*_3,3_ = 15.997, *p* = 0.003). PC4 is driven by mate pair as well (Figure S6A, two‐way ANOVA, 9% variance, *F*_3,3_ = 28.625, *p* < 0.001), but PCs 3 and 5–10 for this stage are all nonsignificant. Pluteus gene expression patterns were largely driven by mate pair, which was significant on both PC1 (34% variance, two‐way ANOVA, *F*_3,3_ = 10.621, *p* = 0.008) and PC2 (13% variance, two‐way ANOVA, *F*_3,3_ = 11.487, *p* = 0.007). For the pluteus stage, treatment did not significantly drive patterns on the first two PC axes. Mate pair was also significant on PC3 (Figure S6B, two‐way ANOVA, 9% variance, *F*_3,3_ = 5.3823, *p* = 0.039) and PC4 (two‐way ANOVA, 7% variance, *F*_3,3_ = 7.787, *p* = 0.017), with a slight effect of treatment on PC4 (two‐way ANOVA, 7% variance, *F*_3,3_ = 4.557, *p* = 0.054). Meanwhile, PCs 5–10 were nonsignificant in this stage.

**FIGURE 5:**
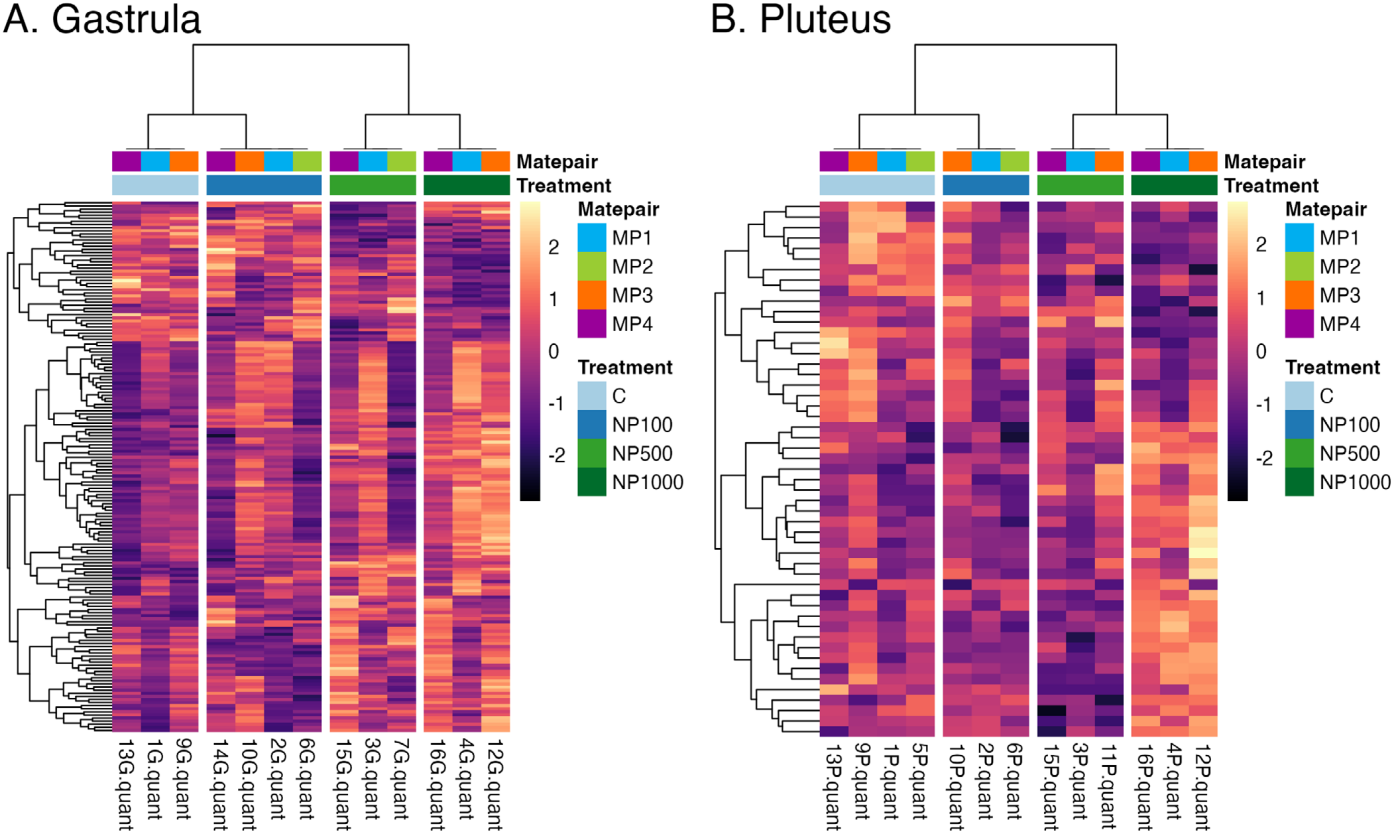
Heat map of top differentially expressed genes among treatments in the gastrula stage (A) and pluteus stage (B). The scale highlights differences in standardized expression levels and how up or downregulated gene expression is relative to the average expression across samples, with increased upregulation shown in light yellow and increased downregulation shown in dark purple. The top row shows the different mate pairs: MP1 (blue), MP2 (green), MP3 (orange), and MP4 (purple). The bottom row shows the different treatments: Control (light blue), 100 ppb nonylphenol (dark blue), 500 ppb nonylphenol (light green), and 1000 ppb nonylphenol (dark green). (A) In the gastrula stage, patterns of upregulation in the nonylphenol treatments compared to the control across mate pairs are apparent. Using a Gene Ontology (GO) database to cross‐reference our identified genes, the 171 DEGs in the gastrula stage were associated with a heat shock protein, ribosomal proteins, embryonic histones, a hepatic leukemia factor, and transcript variants. *p*‐values highlight the differences between the full model, including treatment and mate pair compared to the reduced model with only mate pair. All *p*‐values for differentially expressed genes are included in Table S2. (B) In the pluteus stage, the control and 1000 ppb treatments appear to differ, with genes showing upregulation in the control flipping and showing downregulation in the 1000 ppb treatment. Again using a Gene Ontology (GO) database to cross‐reference our identified genes, the 57 DEGs in the pluteus stage were associated with ribosomal proteins, tubulin‐associated genes, zinc finger protein, and neurotrypsin transcript variants. *p*‐values for all differentially expressed genes included in Table S4.

In the gastrula stage, 171 genes were differentially expressed because of treatment (Likelihood Ratio Test (LRT), *p* < 0.05). In the heat map showing these 171 genes and their scaled expression level, we see upregulation in all of the nonylphenol exposure treatment conditions compared to the control in the gastrula stage, even in the presence of variation between mate pairs (Figure 5A). The topmost differentially expressed gene was a 10 kDa heat shock protein. Expression for this gene was positively correlated with treatment, with the lowest expression in the control and the highest expression in the 1000 ppb treatment (Figure S7A). Other significantly differentially expressed genes were genes associated with ribosomal proteins, embryonic histones, a hepatic leukemia factor, and transcript variants (Table S2). Within the 171 differentially expressed genes, GO enrichment analysis identified 81 overrepresented GO terms (*p* < 0.05). These GO terms were associated with organelle development and metabolic processes (Table S3).

For the pluteus stage, we identified 57 genes that were significantly differentially expressed among treatments (LRT, *p* < 0.05). The heatmap of these genes shows differences between the control and 1000 ppb, but patterns are split between upregulation with treatment and downregulation with treatment (Figure 5B). The topmost differentially expressed gene was a ribosomal protein. This gene had much higher expression in the 1000 ppb treatment than in all other treatments (Figure S7B). Other significantly differentially expressed genes were associated with ribosomal proteins, tubulin‐associated genes, zinc finger protein, and transcript variants (Table S4). GO enrichment analysis identified 34 overrepresented GO terms (*p* < 0.05). These GO terms were associated with genes involved in microtubule processes and skeletal development (Table S5). There were seven shared differentially expressed genes between the two stages. Notably, all the shared genes were associated with ribosomal protein expression (Table 1).

**TABLE 1:** Significantly differentially expressed genes among treatments shared between the gastrula and pluteus developmental stages. All are either transcript variants, associated with ribosomal protein expression or both. The columns indicate transcript name, ensemble gene ID, transcript description and the *p*adjust value for each stage for the shared transcripts that were identified as differentially expressed because of treatment using a likelihood ratio test (LRT) via DeSeq2.

Transcript	ensembl_gene_id	*p*adj gastrula	*p*adj pluteus	Description
XM_030983020	LOC105441189	0.0007	0.050	40S ribosomal protein S21
XM_030979044	LOC592738	0.005	0.030	40S ribosomal protein S24
XM_030973879	LOC577852	9.41E‐06	0.007	60S ribosomal protein L30, transcript variant X2
XM_030994862	LOC587551	0.0008	0.030	60S ribosomal protein L24
XM_030995181	LOC115918936	0.002	0.002	40S ribosomal protein S11‐like, transcript variant X1
XM_788739	LOC589085	0.006	0.031	60S ribosomal protein L31
XM_779989	LOC579897	0.0005	0.050	60S ribosomal protein L35a

## 4 Discussion

Organisms inhabiting the marine intertidal zone along urban coastlines face a complex mixture of stressors, including chemicals originating from stormwater runoff and sewage wastewater pollutants alongside coastline development, fishing pressure, and other stressors (Alter et al. 2021). Understanding how land‐based pollutants impact early life stages of marine invertebrates is important for understanding the long‐term effects of pollution on populations living in these marine urban environments. In this study, we examined the effects of three concentrations of the most common urban‐associated alkylphenol, nonylphenol, on Pacific purple sea urchin larvae. We find no effect of nonylphenol on the earliest developmental stages, but we began to see inconsistent patterns of survival, morphology, and developmental abnormalities between mate pairs starting at the blastula stage. Gene expression data suggest a role for ribosomal pathways in response to nonylphenol treatment. Interestingly, all development, morphological, and transcriptomic data were strongly affected by mate pair. Combined, our work shows that EDCs can have strong effects on early developmental stages, but that effect might vary across larvae from different crosses, even within the same population.

### 4.1 Variation in Developmental Effects of Nonylphenol Among Mate Pairs

In our survival data, we observed the strongest decrease in survival at the lowest concentration of nonylphenol (one‐sample *t*‐test, *p* = 0.060). This is not an uncommon finding in ecotoxicology studies with endocrine disrupting chemicals. These chemicals often exhibit a non‐conforming dose–response curve, or a nonmonotonic response, which means they can have greater impacts at lower concentrations than higher concentrations (Vandenberg 2014). A similar phenomenon was observed in *S. purpuratus* larvae exposed to plastic‐associated chemicals (Shore et al. 2022) and a different sea urchin species, *Paracentrotus lividus* , exposed to nonylphenol (Arslan et al. 2007). Neither our study nor the examples cited directly addressed the mechanism behind the observed nonmonotonic responses, and more work is needed to investigate if different concentrations of nonylphenol and other EDC exposure result in shifts in endocrine hormones alongside the developmental and transcriptomic effects. Other invertebrates, such as the nematode *Caenorhabditis elegans* , showed the biggest impact on growth at the lowest concentrations of nonylphenol, suggesting that low, ecologically relevant concentrations can still have a large impact on body size (De La Parra‐Guerra et al. 2020).

Effects of nonylphenol vary both across life history and among mate pairs. The earliest developmental stages showed no effects of nonylphenol treatment; fertilization and development success were consistently high across all treatment concentrations and families. Similar findings of high fertilization and early development were reported in another study where Pacific purple sea urchin larvae were exposed to plastic additives (Shore et al. 2022). One possible explanation for this observation is that prior to the blastula stage (24 hpf), embryos rely on maternal transcripts, but once they reach the blastula stage, they begin expressing their own transcriptomes (Garfield et al. 2013). Adult exposure has even been shown to influence the impacts of EDCs on offspring, with maternal exposure resulting in less sensitive developing embryos (Roepke et al. 2006). In our data, we begin to see effects of treatment at the blastula stage when embryos begin expressing their own genes and are quickly developing at only 24 h postfertilization. After the blastula stage, we see effects of treatment on morphology, but that effect varies dramatically across mate pairs. For example, gastrula height varied across treatments but showed different trajectories in different mate pairs. Gastrula height has been shown to significantly decrease with elevated *p*CO_2_ levels (Wong and Hofmann 2020), suggesting that stressful conditions may result in smaller gastrula as a compensation mechanism, but our data also highlight that different mate pairs can have even opposing morphological differences across stressful conditions. Similarly, we did not find any statistically significant effects of treatment on the proportion of abnormalities, but different families appeared to have very different reaction norms in response to different concentrations of nonylphenol. Genotype differences between families have been shown to affect larval responses in several marine invertebrate species when exposed to changing temperatures or ocean acidification, with certain mate pairs better able to tolerate environmental stress than others (Delorme and Sewell 2016; Foo et al. 2012; Foo and Byrne 2017). Although we do not have genotype information for our individuals used in this study, it is assumed they are closely related because of all individuals coming from the same population and the significant lack of population structure across all of California in this species (Rumberger et al. 2025).

The observation of dramatic variation across mate pairs is an important consideration for future developmental stress response studies. Many studies designed to understand the response to stressors at early life stages either use a single cross (Shore et al. 2022; Uibel 2016) or pool gametes or larvae (Roepke et al. 2005; Wong and Hofmann 2020). Our results highlight the diversity of responses possible among different mate pairs; even the directionality of response occasionally differed among our different crosses. Additionally, some responses are most appropriately interpreted in the context of other, mate pair‐specific responses. For example, mate pair 2 had extremely low survival at the highest concentrations of nonylphenol. Therefore, the apparent low proportion of abnormality of this pair at high nonylphenol concentrations might just be a result of low sample sizes and the death of any abnormal larvae, rather than actual resistance to treatment. More research is needed with larger sample sizes and more mate pairs, however, to disentangle this phenomenon. The variation among pairs is perhaps not surprising considering the strong effect of genotype on physiology more broadly (Delorme and Sewell 2016; Foo et al. 2012; Foo and Byrne 2017). Although our experiment did not have the within‐family replication to explicitly test this response diversity, future studies should explicitly integrate potential genetic effects into the study design.

### 4.2 Gene Expression Associated With Treatment

Like other measures of performance, our gene expression patterns group mainly by mate pair (Figure 4). Gene expression can be heavily influenced by parental genetics, which may account for the differences in response to nonylphenol even from urchins in the same population (Devens et al. 2020). However, we were also able to uncover differential expression among treatments. The top differentially expressed gene identified in the gastrula stage was a heat shock protein (HSP), which has been shown to be a generalized stress response and has been shown to be upregulated during stress in Pacific purple urchin larvae ( *Strongylocentrotus purpuratus* , Hamdoun and Epel 2007; Hamdoun et al. 2004) and mussels ( *Mytilus galloprovincialis* , Balbi et al. 2016) exposed to BPA. HSPs have also been identified as a stress response under EDC exposure in a wide range of other species, including *C. elegans* , *Drosophila*, marine crabs ( *Charybdis japonica* ), shrimps ( *Litopenaeus vannamei* ), moths (*Sesamia nonagrioides*), and zebrafish (De La Parra‐Guerra et al. 2020; Dwivedi et al. 2022; Jia et al. 2016; Michail et al. 2012; Park and Kwak 2014; Su et al. 2024). Marine crabs ( *Charybdis japonica* ) showed increased heat shock protein expression with increasing concentrations of nonylphenol, with the highest expression in the ovaries, gill, and pancreas tissues (Park and Kwak 2014). Genes responding to nonylphenol treatment across both developmental stages were exclusively ribosomal genes (Table 1). Downregulation in ribosomal protein expression in response to EDC exposure has been observed in *Saccharomyces* (Bereketoglu et al. 2017), whereas the opposite pattern has been observed in medaka fish ( *Oryzias javanicus* ) (Won et al. 2014), suggesting increasing levels of translation.

## 5 Conclusion

Urbanization exposes organisms to complex and novel stressors. Urban ecology and evolution is a rapidly developing field (Des Roches et al. 2021; Diamond and Martin 2021; Johnson and Munshi‐South 2017; Lambert et al. 2021), and the marine intertidal zone is an emerging zone of interest because of the unique pathways of exposure. Our study identified that treatment effects from nonylphenol do not materialize until after the blastula stage, and effects varied greatly by mate pair. In addition, effects on survival were observed even at the lowest concentration. We identify important genes and pathways responding to nonylphenol exposure, which can inform future research on molecular mechanisms and biomarker development. Future studies should focus on understanding variation in response among individuals and populations, allowing this ecotoxicology response to be placed in an eco‐evolutionary framework and integrated into predictions of population response to urbanization.

## Author Contributions

**Madison L. Armstrong:** conceptualization (lead), data curation (lead), formal analysis (lead), funding acquisition (lead), investigation (lead), methodology (lead), visualization (equal), writing – original draft (equal), writing – review and editing (equal). **Sindhu Bala:** conceptualization (supporting), formal analysis (supporting), funding acquisition (supporting), investigation (equal), methodology (supporting), visualization (equal), writing – original draft (equal), writing – review and editing (equal). **Rachael A. Bay:** resources (equal), supervision (equal), writing – review and editing (equal).

## Conflicts of Interest

The authors declare no conflicts of interest.

## Supporting information


**Data S1:** ece372183‐sup‐0001‐supinfo.docx.

## Data Availability

Data and scripts are uploaded on GitHub: https://github.com/mlarmstrong/exposure_urch. Raw sequence data for transcriptomics analyses are on NCBI: Accession PRJNA1288155.
