# Supplementary material for: Developmental and Transcriptomic Responses in Sea Urchin Larvae to an Urban‐Associated Pollutant
Source: Ecol Evol. 2025 Sep 17;15(9):e72183. doi: 10.1002/ece3.72183 (PMC12443524; doi:10.1002/ece3.72183)
Supplement: Supplementary file 1 — Data S1: ece372183‐sup‐0001‐supinfo.docx. [file ECE3-15-e72183-s001.docx]

**
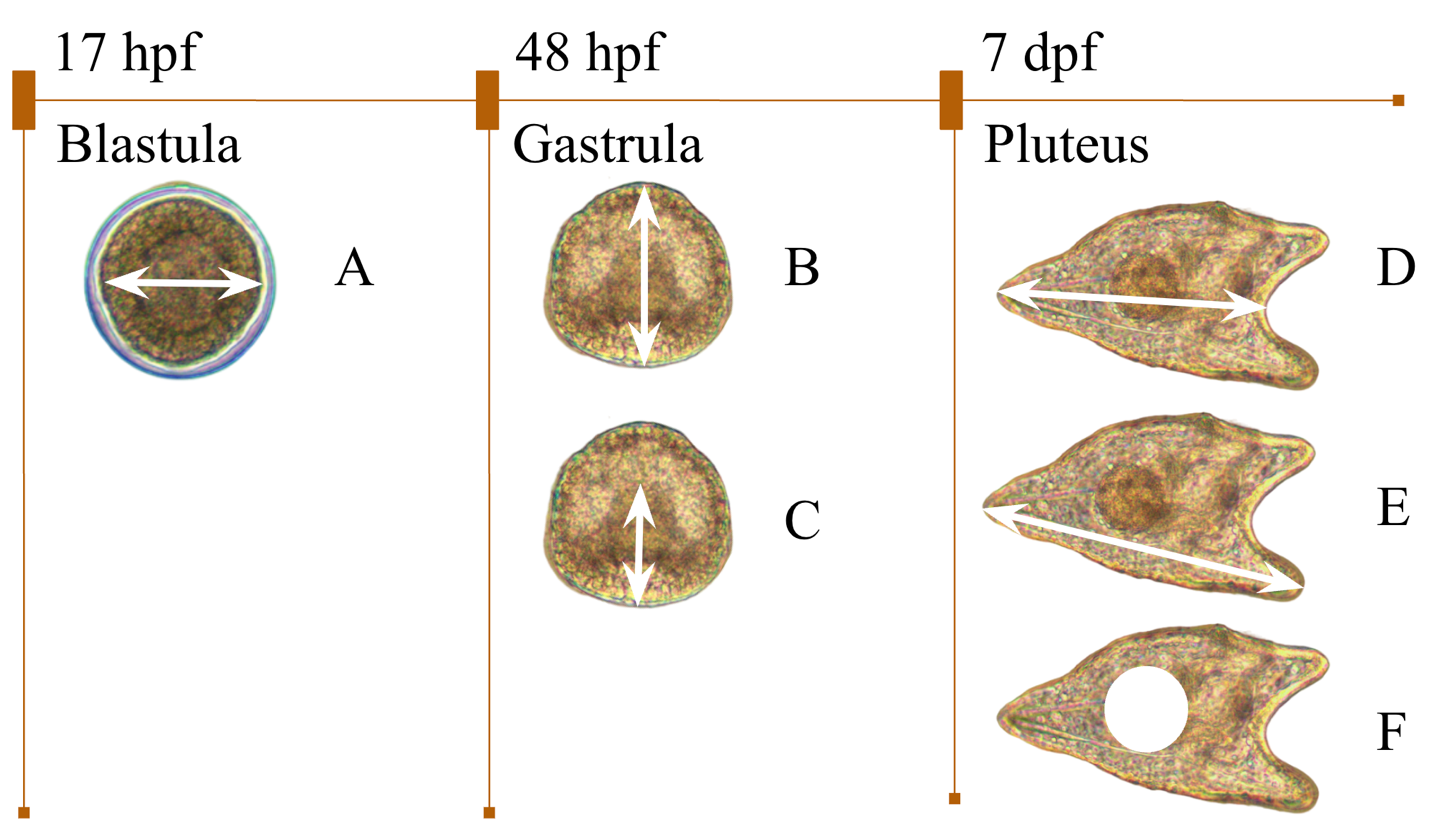
**

***Supplemental Figure 1: Measurements taken at each developmental stage.*** *At the blastula stage, the embryo’s diameter was measured (A). At the gastrula stage, height (B) and stomach length (C) were measured. At the pluteus stage, body length (D), arm length (E), and stomach area (F) were measured.*

**
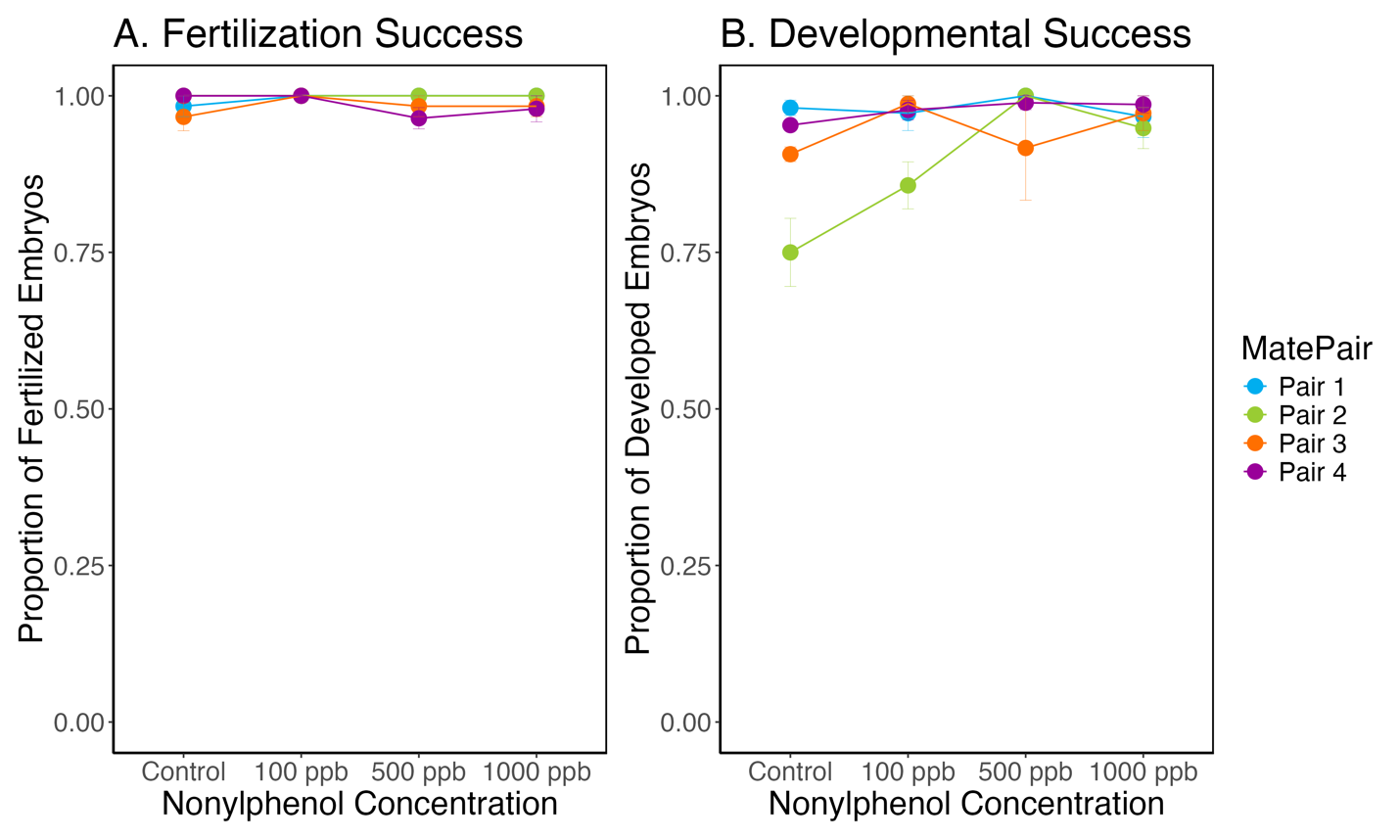
Supplemental** **Figure 2: Fertilization and developmental success data across nonylphenol treatments.** Different colors represent the four mate pairs. Error bars for each point represent standard error. No impact of treatment on fertilization and development success was observed. We saw no significant difference in fertilization success between treatments (two-way ANOVA, F_3,3_= 1.0653, p=0.4111) or across mate pairs (two-way ANOVA, F_3,3_= 1.8349, p=0.0.2110). A similar pattern was seen for development success, with no significant differences between treatments (two-way ANOVA, F_3,3_= 1.644, p=0.247) or across mate pairs (two-way ANOVA, F_3,3_= 2.333, p=0.142).

**Supplemental Table 1:** Summarized morphology data and statistics for the blastula, gastrula and pluteus stages. The significance of treatment in supplemental figures 2-4 may be driven by the extremely large number of larvae measured per beaker (blastula: mean=56 embryos, range=0-93; gastrula: mean=33 embryos, range=1-73 ; pluteus: mean=18 larvae, range=0-56).

| Stage | Measurement | Treatment | MatePair | Mean | Standard Deviation |
| --- | --- | --- | --- | --- | --- |
| Blastula | Blastula_height | Control | 1 | 0.087 | 0.003 |
| Blastula | Blastula_height | 100ppb | 1 | 0.093 | 0.005 |
| Blastula | Blastula_height | 500ppb | 1 | 0.094 | 0.004 |
| Blastula | Blastula_height | 1000ppb | 1 | 0.097 | 0.005 |
| Blastula | Blastula_height | Control | 2 | 0.093 | 0.003 |
| Blastula | Blastula_height | 100ppb | 2 | 0.103 | 0.007 |
| Blastula | Blastula_height | 500ppb | 2 | 0.093 | 0.003 |
| Blastula | Blastula_height | Control | 3 | 0.086 | 0.004 |
| Blastula | Blastula_height | 100ppb | 3 | 0.093 | 0.003 |
| Blastula | Blastula_height | 500ppb | 3 | 0.093 | 0.003 |
| Blastula | Blastula_height | 1000ppb | 3 | 0.098 | 0.005 |
| Blastula | Blastula_height | Control | 4 | 0.089 | 0.003 |
| Blastula | Blastula_height | 100ppb | 4 | 0.087 | 0.004 |
| Blastula | Blastula_height | 500ppb | 4 | 0.085 | 0.003 |
| Blastula | Blastula_height | 1000ppb | 4 | 0.083 | 0.003 |
| Gastrula | Gastrula_height | Control | 1 | 0.120 | 0.012 |
| Gastrula | Gastrula_height | 100ppb | 1 | 0.119 | 0.016 |
| Gastrula | Gastrula_height | 500ppb | 1 | 0.115 | 0.007 |
| Gastrula | Gastrula_height | 1000ppb | 1 | 0.115 | 0.023 |
| Gastrula | Gastrula_stomachlength | Control | 1 | 0.084 | 0.017 |
| Gastrula | Gastrula_stomachlength | 100ppb | 1 | 0.090 | 0.016 |
| Gastrula | Gastrula_stomachlength | 500ppb | 1 | 0.085 | 0.014 |
| Gastrula | Gastrula_stomachlength | 1000ppb | 1 | 0.096 | 0.011 |
| Gastrula | Gastrula_height | Control | 2 | 0.131 | 0.008 |
| Gastrula | Gastrula_height | 100ppb | 2 | 0.135 | 0.010 |
| Gastrula | Gastrula_height | 500ppb | 2 | 0.123 | 0.009 |
| Gastrula | Gastrula_stomachlength | Control | 2 | 0.100 | 0.013 |
| Gastrula | Gastrula_stomachlength | 100ppb | 2 | 0.099 | 0.016 |
| Gastrula | Gastrula_stomachlength | 500ppb | 2 | 0.093 | 0.010 |
| Gastrula | Gastrula_height | Control | 3 | 0.111 | 0.018 |
| Gastrula | Gastrula_height | 100ppb | 3 | 0.119 | 0.005 |
| Gastrula | Gastrula_height | 500ppb | 3 | 0.113 | 0.008 |
| Gastrula | Gastrula_height | 1000ppb | 3 | 0.121 | 0.008 |
| Gastrula | Gastrula_stomachlength | Control | 3 | 0.070 | 0.012 |
| Gastrula | Gastrula_stomachlength | 100ppb | 3 | 0.064 | 0.007 |
| Gastrula | Gastrula_stomachlength | 500ppb | 3 | 0.067 | 0.008 |
| Gastrula | Gastrula_stomachlength | 1000ppb | 3 | 0.082 | 0.018 |
| Gastrula | Gastrula_height | Control | 4 | 0.111 | 0.007 |
| Gastrula | Gastrula_height | 100ppb | 4 | 0.096 | 0.009 |
| Gastrula | Gastrula_height | 500ppb | 4 | 0.117 | 0.006 |
| Gastrula | Gastrula_height | 1000ppb | 4 | 0.118 | 0.007 |
| Gastrula | Gastrula_stomachlength | Control | 4 | 0.083 | 0.013 |
| Gastrula | Gastrula_stomachlength | 100ppb | 4 | 0.063 | 0.006 |
| Gastrula | Gastrula_stomachlength | 500ppb | 4 | 0.085 | 0.014 |
| Gastrula | Gastrula_stomachlength | 1000ppb | 4 | 0.090 | 0.012 |
| Pluteus | Pluteus_bodylength | Control | 1 | 0.241 | 0.022 |
| Pluteus | Pluteus_bodylength | 100ppb | 1 | 0.254 | 0.022 |
| Pluteus | Pluteus_bodylength | 500ppb | 1 | 0.238 | 0.024 |
| Pluteus | Pluteus_bodylength | 1000ppb | 1 | 0.246 | 0.015 |
| Pluteus | Pluteus_armlength | Control | 1 | 0.305 | 0.035 |
| Pluteus | Pluteus_armlength | 100ppb | 1 | 0.318 | 0.034 |
| Pluteus | Pluteus_armlength | 500ppb | 1 | 0.299 | 0.033 |
| Pluteus | Pluteus_armlength | 1000ppb | 1 | 0.318 | 0.023 |
| Pluteus | Pluteus_stomacharea | Control | 1 | 0.004 | 0.001 |
| Pluteus | Pluteus_stomacharea | 100ppb | 1 | 0.005 | 0.001 |
| Pluteus | Pluteus_stomacharea | 500ppb | 1 | 0.004 | 0.001 |
| Pluteus | Pluteus_stomacharea | 1000ppb | 1 | 0.004 | 0.001 |
| Pluteus | Pluteus_bodylength | Control | 2 | 0.241 | 0.018 |
| Pluteus | Pluteus_bodylength | 100ppb | 2 | 0.268 | 0.017 |
| Pluteus | Pluteus_bodylength | 500ppb | 2 | 0.239 | 0.012 |
| Pluteus | Pluteus_armlength | Control | 2 | 0.313 | 0.023 |
| Pluteus | Pluteus_armlength | 100ppb | 2 | 0.347 | 0.030 |
| Pluteus | Pluteus_armlength | 500ppb | 2 | 0.299 | 0.020 |
| Pluteus | Pluteus_stomacharea | Control | 2 | 0.004 | 0.001 |
| Pluteus | Pluteus_stomacharea | 100ppb | 2 | 0.004 | 0.001 |
| Pluteus | Pluteus_stomacharea | 500ppb | 2 | 0.003 | 0.001 |
| Pluteus | Pluteus_bodylength | Control | 3 | 0.223 | 0.019 |
| Pluteus | Pluteus_bodylength | 1000ppb | 3 | 0.237 | 0.023 |
| Pluteus | Pluteus_armlength | Control | 3 | 0.266 | 0.025 |
| Pluteus | Pluteus_armlength | 1000ppb | 3 | 0.289 | 0.020 |
| Pluteus | Pluteus_stomacharea | Control | 3 | 0.004 | 0.001 |
| Pluteus | Pluteus_stomacharea | 1000ppb | 3 | 0.004 | 0.001 |
| Pluteus | Pluteus_bodylength | Control | 4 | 0.244 | 0.018 |
| Pluteus | Pluteus_bodylength | 500ppb | 4 | 0.226 | 0.023 |
| Pluteus | Pluteus_bodylength | 1000ppb | 4 | 0.246 | 0.018 |
| Pluteus | Pluteus_armlength | Control | 4 | 0.286 | 0.021 |
| Pluteus | Pluteus_armlength | 500ppb | 4 | 0.267 | 0.028 |
| Pluteus | Pluteus_armlength | 1000ppb | 4 | 0.288 | 0.022 |
| Pluteus | Pluteus_stomacharea | Control | 4 | 0.004 | 0.001 |
| Pluteus | Pluteus_stomacharea | 500ppb | 4 | 0.003 | 0.001 |
| Pluteus | Pluteus_stomacharea | 1000ppb | 4 | 0.003 | 0.001 |


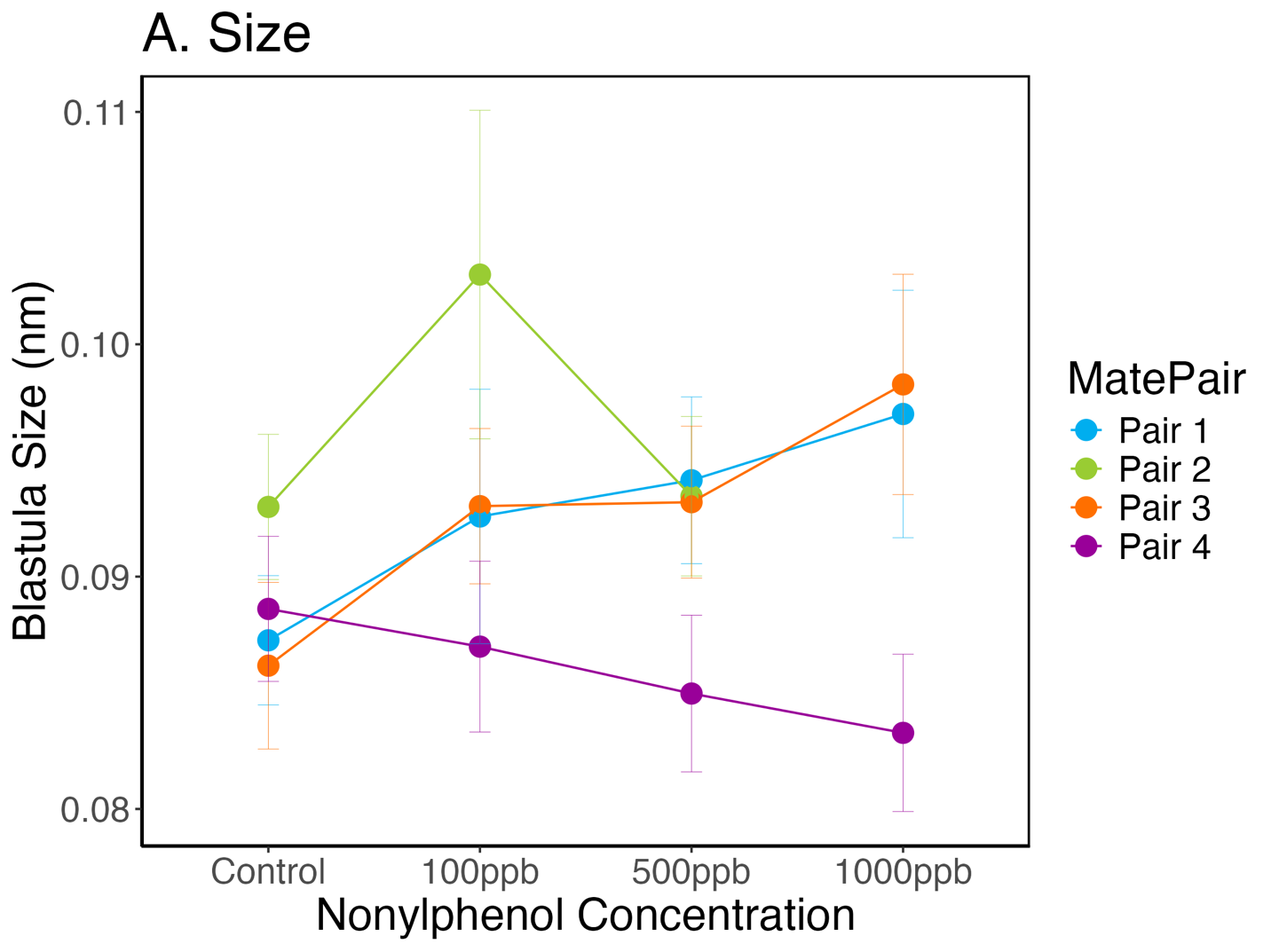


**Supplemental Figure 3: Blastula size data across nonylphenol concentrations.** Different colors represent different mate pairs. Size was strongly determined by mate pair and there were inconsistent patterns across treatments. Both mate pair (two-way ANOVA, F_3,895_ =233.349, p<0.001) and treatment (two-way ANOVA, F_3,_ _898_=77.118, p<0.001) were significantly impacted blastula size, but this is likely due to the strong interactive effect mate pair had with treatment in this morphological measurement (two-way ANOVA, F_8,_ _887_=62.591, p<0.001).


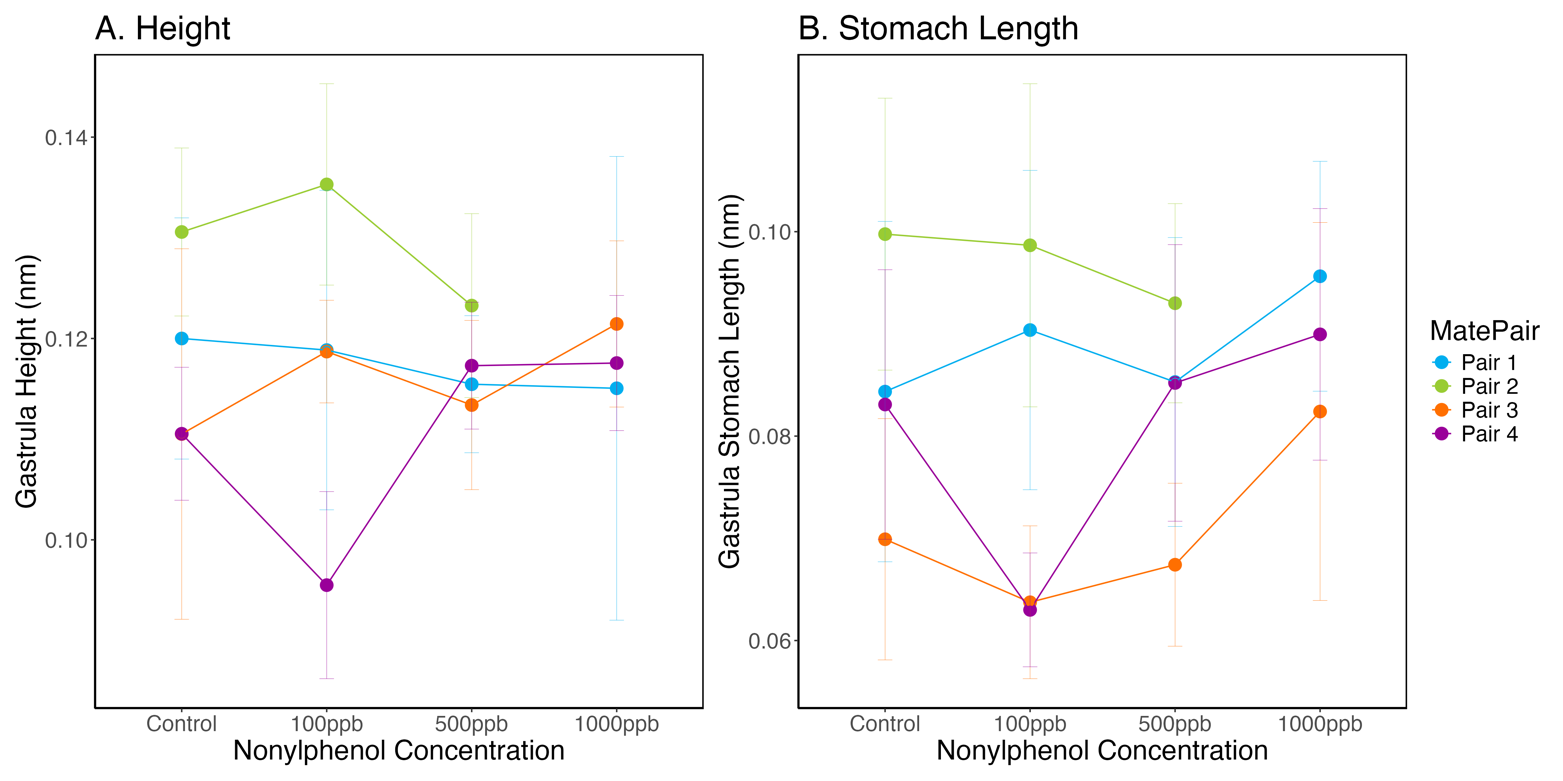


**Supplemental Figure 4:** **Gastrula morphological data: size/height (A) and stomach length (B).**  Different colors represent different mate pairs. Variation in gastrula height was strongly driven by mate pair differences (two-way ANOVA, F_3,575_ =39.0422, p<0.001) and treatment (two-way ANOVA, F_3,_ _578_=6.5964, p<0.001), but this is likely due to the strong interactive effect mate pair had with treatment (two-way ANOVA, F_9,_ _566_=4.4324, p<0.001). A similar pattern was shown in gastrula stomach length, with variation driven by both mate pair (two-way ANOVA, F_3,_ _421_=70.4460, p<0.001) and treatment (two-way ANOVA, F_3,_ _423_=5.4415, p=0.001119), but a strong impact due to the interaction of the two (two-way ANOVA, F_9,_ _4112_=4.3769, p<0.001).


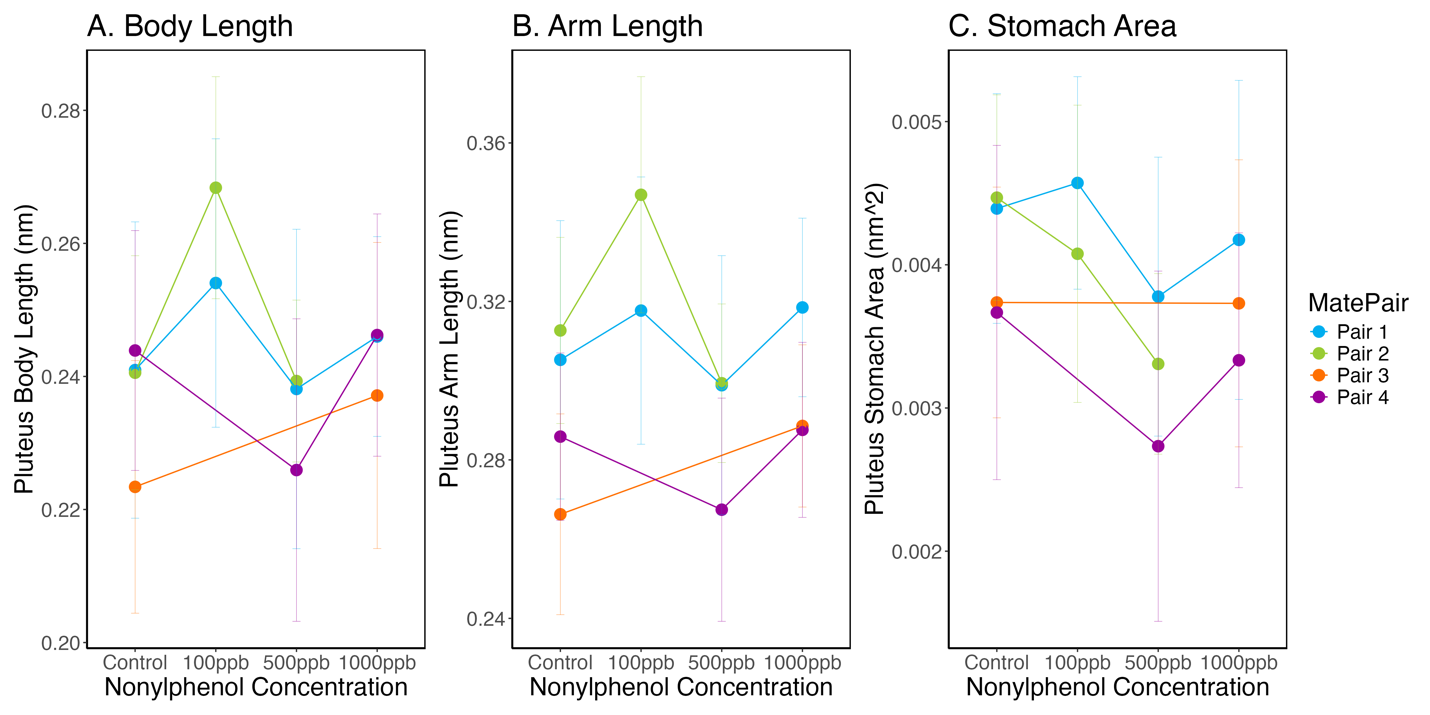


**Supplemental Figure 5: Pluteus morphological data: body length (A), arm length (B) and stomach area (C).** Different colors show different mate pairs. A similar pattern from the blastula and gastrula data was observed in the pluteus morphological data, with size at all three measurements varying by mate pair, treatment and the interaction between treatment and mate pair. For body length, the interaction was not as strong (two-way ANOVA, F_7,_ _267_=2.2856, p=0.0281784) as mate pair (two-way ANOVA, F_3,_ _274_=6.4272, p<0.001) and treatment (two-way ANOVA, F_3,_ _277_=9.3869, p<0.001) alone. For arm length, only treatment alone (two-way ANOVA, F_3,_ _276_=11.626, p<0.001) and mate pair alone (two-way ANOVA, F_3,_ _273_=25.103, p<0.001) were significant, with no interactive effect (two-way ANOVA, F_7,_ _266_=1.663, p=0.1183). Lastly for pluteus stomach area, we saw only an effect of treatment (two-way ANOVA, F_3,_ _307_=15.1545, p<0.001) and mate pair (two-way ANOVA, F_3,_ _304_=6.5964, p<0.001) but no interaction (two-way ANOVA, F_7,_ _297_=0.7943, p=0.5926).


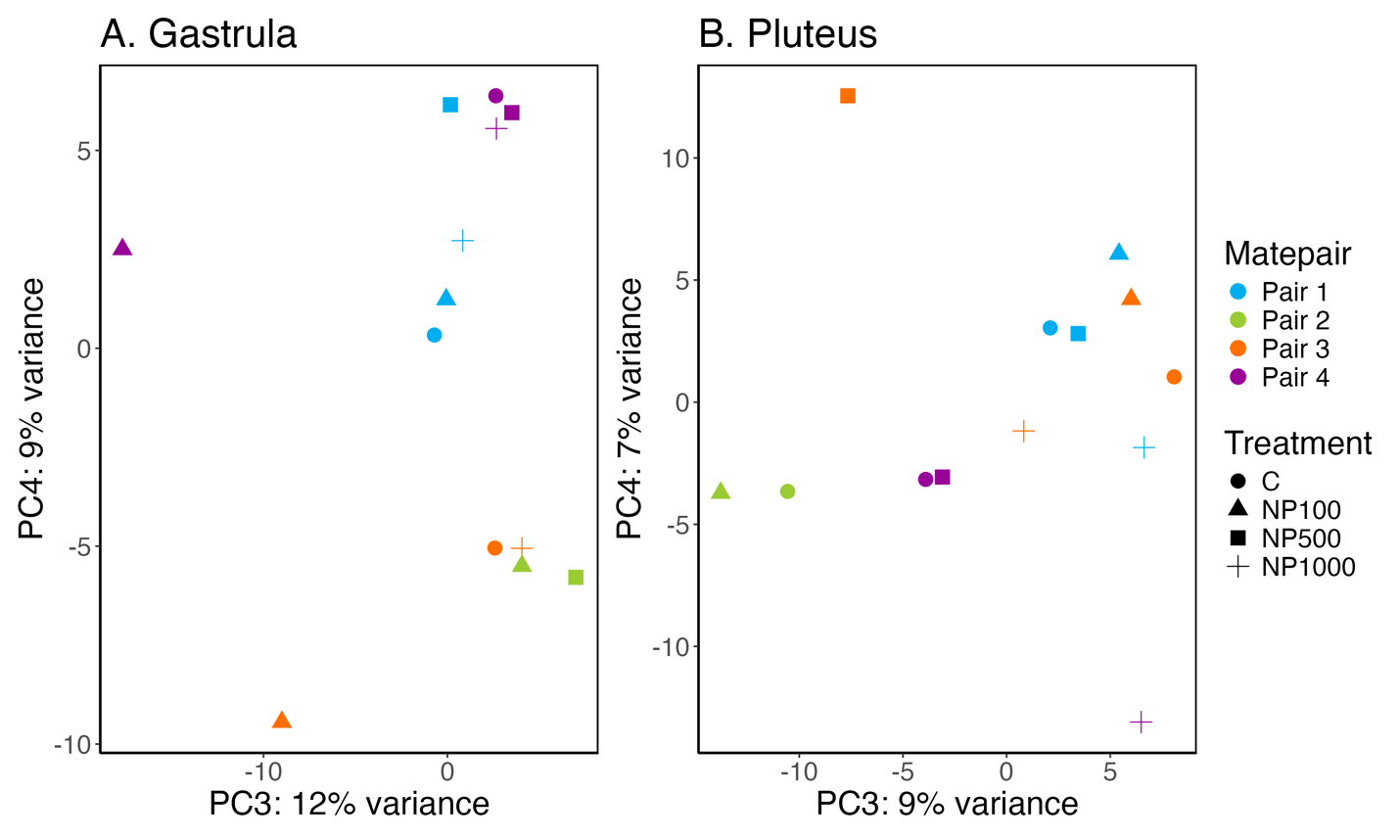


***Supplemental Figure 6: Additional PC axes for gene expression samples with mate pair (colors) and treatments (shapes) across two developmental stages, gastrula (A) and pluteus (B).*** *(6A): For the gastrula stage, PC4 is driven by mate pair as well (two-way ANOVA, 9% variance, F_3,3_= 28.625, p<0.001), but PCs 3 and 5-10 for this stage are all nonsignificant. (6B) For the pluteus stage, mate pair was also significant on PC3 (two-way ANOVA, 9% variance, F_3,3_= 5.3823, p=0.039) and PC4 (two-way ANOVA, 7% variance, F_3,3_=7.787, p=0.017), with a slight effect of treatment on PC4 (two-way ANOVA, 7% variance, F_3,3_= 4.557, p=0.054). Meanwhile PCs 5-10 were nonsignificant in this stage.*

*
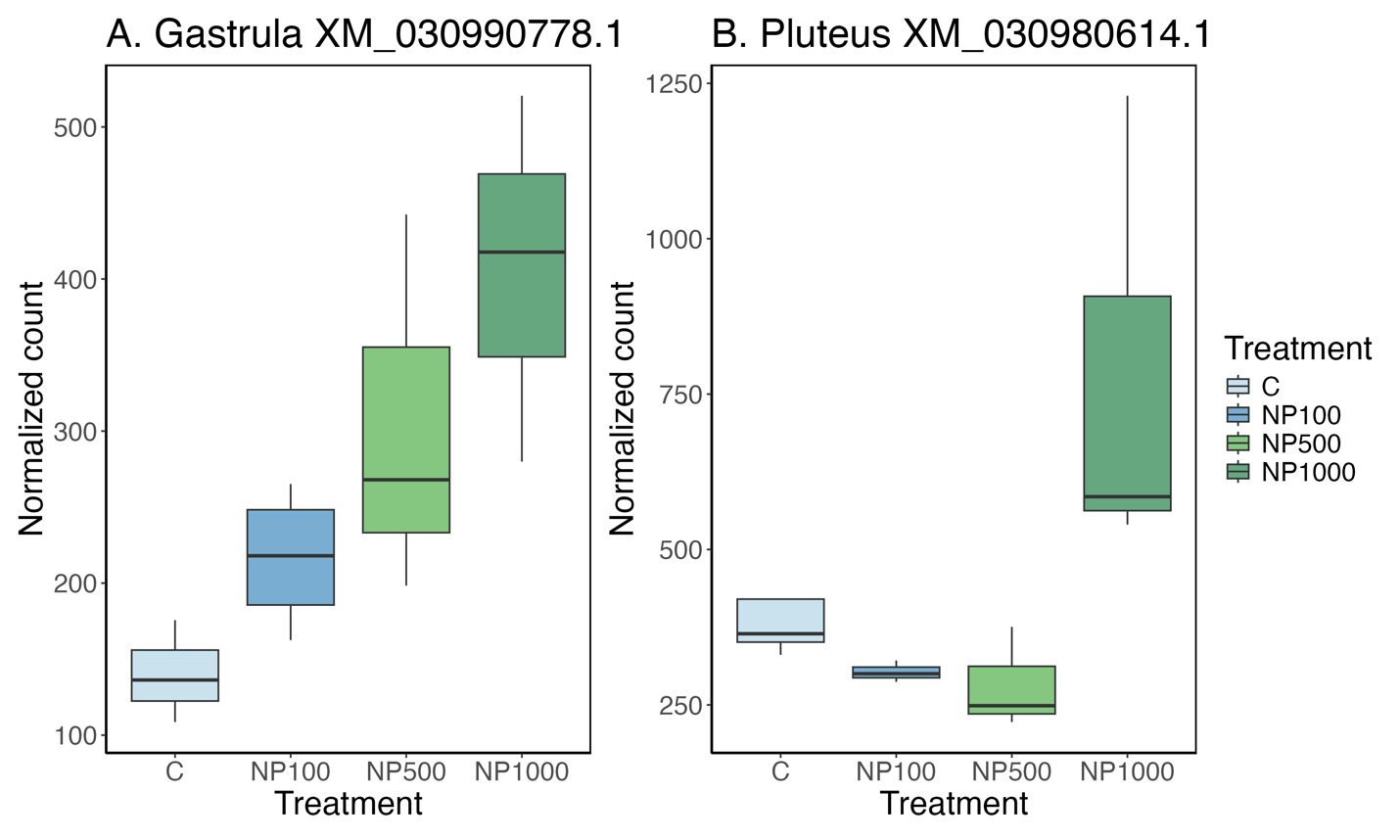
*

***Supplemental Figure 7: Normalized counts from the Likelihood Ratio Test (LRT) analysis via DeSeq2 (Love et al., 2014) for the top differentially expressed gene in the gastrula (A) and pluteus (B) developmental stages.*** *Colors correspond to the different treatments: Control (C, light blue), 100ppb nonylphenol (NP100, blue), 500ppb nonylphenol (NP500, light green) and 1000ppb nonylphenol (NP1000, dark green). The y-axis shows normalized counts. (A) The top differentially expressed gene in the gastrula stage was a 10 kDa heat shock protein (Transcript ID: XM_030990778.1, Ensembl Gene ID: LOC762428). The highest counts were in the 1000ppb nonylphenol treatment and the lowest was in the control, while the 500ppb nonylphenol treatment (p=0.05) and the 1000ppb nonylphenol treatment (p=0.005) significantly differed from the control. (B)The top differentially expressed gene in the pluteus stage was a 60S ribosomal protein (Transcript ID: XM_030980614.1, Ensembl Gene ID: LOC115922215). The highest counts were in the 1000ppb nonylphenol treatment, with high variance between mate pairs, while the lowest were in the 500ppb nonylphenol treatment (Supplemental Figure 7B). Additionally, the 1000ppb nonylphenol treatment also significantly differed from the control (p=0.033).*

***Supplemental Table 2 :*** *Significantly differentially expressed genes (171) identified with a likelihood ratio test (LRT) via DeSeq2 (Love et al., 2014) between treatments the gastrula stage. Columns show transcript name, the statistical comparison of expression between treatments (stat), the p-value, p-adjust value, gene ID and description. P-values highlight the differences between the full model with mate pair and treatment compared to the reduced model with only mate pair. Using gene ontology to identify gene function, the topmost differentially expressed gene was associated with a 10kDa heat shock protein. Other significantly differentially expressed genes were associated with ribosomal proteins, embryonic histones, a hepatic leukemia factor and transcript variants.*

| Transcript | stat | pvalue | padj | ensembl_gene_id | description |
| --- | --- | --- | --- | --- | --- |
| XM_030990778.1 | 71.574 | 1.96E-15 | 3.49E-12 | LOC762428 | 10 kDa heat shock protein, mitochondrial |
| XR_143688.3 | 52.546 | 2.29E-11 | 2.03E-08 | LOC575048 | uncharacterized LOC575048, transcript variant X2 |
| XM_030992705.1 | 51.602 | 3.64E-11 | 2.15E-08 | LOC115927200 | paraneoplastic antigen Ma2 homolog |
| XM_030973206.1 | 43.666 | 1.78E-09 | 7.88E-07 | LOC578794 | 40S ribosomal protein S27-like, transcript variant X2 |
| XR_004065522.1 | 43.085 | 2.36E-09 | 8.38E-07 | LOC115927253 | uncharacterized LOC115927253 |
| XR_004063937.1 | 41.421 | 5.32E-09 | 1.58E-06 | LOC115923512 | uncharacterized LOC115923512 |
| XR_004063973.1 | 39.708 | 1.23E-08 | 3.12E-06 | LOC115923586 | uncharacterized LOC115923586, transcript variant X2 |
| XR_004065386.1 | 38.214 | 2.55E-08 | 5.65E-06 | LOC115917849 | U1 spliceosomal RNA |
| XM_003726257.3 | 37.797 | 3.12E-08 | 6.16E-06 | LOC100891486 | membrane magnesium transporter 1 |
| XM_030973879.1 | 36.709 | 5.30E-08 | 9.41E-06 | LOC577852 | 60S ribosomal protein L30, transcript variant X2 |
| NM_001129814.1 | 33.244 | 2.86E-07 | 4.62E-05 | GeneID_593063 | hepatic leukemia factor |
| XR_973839.2 | 31.910 | 5.47E-07 | 8.02E-05 | LOC754314 | uncharacterized LOC754314 |
| XR_972005.2 | 31.596 | 6.37E-07 | 8.02E-05 | LOC105441402 | uncharacterized LOC105441402 |
| XM_778407.5 | 31.468 | 6.77E-07 | 8.02E-05 | LOC578220 | dolichyl-diphosphooligosaccharide--protein glycosyltransferase subunit dad1, transcript variant X2 |
| XM_030989541.1 | 31.671 | 6.14E-07 | 8.02E-05 | LOC105441936 | tryparedoxin-like |
| XR_004062287.1 | 30.737 | 9.66E-07 | 0.0001 | LOC115917699 | U1 spliceosomal RNA |
| NM_001242650.1 | 30.352 | 1.16E-06 | 0.0001 | GeneID_574853 | ribosomal protein L36 |
| XR_004064590.1 | 29.719 | 1.58E-06 | 0.0002 | LOC100890329 | bcl-2-like protein 1, transcript variant X4 |
| XM_001176097.4 | 29.334 | 1.91E-06 | 0.0002 | LOC576727 | protein transport protein Sec61 subunit beta |
| XM_030976377.1 | 27.538 | 4.54E-06 | 0.0004 | LOC115920524 | uncharacterized LOC115920524 |
| XM_779989.5 | 27.095 | 5.62E-06 | 0.0005 | LOC579897 | 60S ribosomal protein L35a |
| XM_030995170.1 | 26.554 | 7.30E-06 | 0.0006 | LOC115928210 | c-Myc-binding protein-like |
| XM_030983020.1 | 26.082 | 9.17E-06 | 0.0007 | LOC105441189 | 40S ribosomal protein S21 |
| XM_030994862.1 | 25.610 | 1.15E-05 | 0.001 | LOC587551 | 60S ribosomal protein L24 |
| XM_030992723.1 | 25.279 | 1.35E-05 | 0.001 | LOC579559 | 60S ribosomal protein L12 |
| XM_782899.4 | 25.359 | 1.30E-05 | 0.001 | LOC582971 | barrier-to-autointegration factor, transcript variant X2 |
| XM_011684848.2 | 25.290 | 1.34E-05 | 0.001 | LOC582704 | late histone H2B.2.2-like |
| XM_030989696.1 | 24.275 | 2.19E-05 | 0.001 | LOC581383 | vesicular glutamate transporter 1 |
| NM_214642.1 | 23.788 | 2.77E-05 | 0.002 | LOC373518 | primary mesenchyme specific protein MSP130-related-2 |
| XM_030986658.1 | 23.584 | 3.05E-05 | 0.002 | LOC762974 | translocon-associated protein subunit gamma |
| XM_791421.5 | 23.272 | 3.54E-05 | 0.002 | LOC591874 | 60S ribosomal protein L9 |
| XR_004063667.1 | 23.115 | 3.82E-05 | 0.002 | LOC115922899 | large subunit ribosomal RNA |
| XM_777826.5 | 23.052 | 3.94E-05 | 0.002 | LOC577611 | ER membrane protein complex subunit 3 |
| XR_004062315.1 | 22.935 | 4.17E-05 | 0.002 | LOC115917714 | U1 spliceosomal RNA |
| XR_004065900.1 | 22.594 | 4.91E-05 | 0.002 | LOC115928123 | uncharacterized LOC115928123, transcript variant X2 |
| XM_030995181.1 | 22.645 | 4.79E-05 | 0.002 | LOC115918936 | 40S ribosomal protein S11-like, transcript variant X1 |
| XM_030981575.1 | 22.510 | 5.11E-05 | 0.003 | LOC115922553 | histone H2A, embryonic |
| XM_030972759.1 | 22.269 | 5.73E-05 | 0.003 | LOC590530 | 40S ribosomal protein S23 |
| XR_971289.2 | 21.792 | 7.21E-05 | 0.003 | LOC579768 | subtilisin-chymotrypsin inhibitor-2A, transcript variant X2 |
| XM_011678787.2 | 21.681 | 7.60E-05 | 0.003 | LOC580957 | 60S ribosomal protein L27 |
| XM_790334.5 | 21.684 | 7.59E-05 | 0.003 | LOC590740 | 40S ribosomal protein S12 |
| XM_011675026.2 | 21.315 | 9.06E-05 | 0.004 | LOC579345 | cytochrome c oxidase subunit 5A, mitochondrial, transcript variant X1 |
| XM_791638.5 | 21.267 | 9.27E-05 | 0.004 | LOC592098 | 60S ribosomal protein L18a |
| XM_787297.5 | 21.304 | 9.10E-05 | 0.004 | LOC587578 | cytochrome c oxidase subunit 7A1, mitochondrial |
| NM_001123497.1 | 21.069 | 0.0001 | 0.004 | LOC548620 | elongation factor 1 alpha |
| XR_970749.2 | 20.999 | 0.0001 | 0.004 | LOC105437889 | uncharacterized LOC105437889 |
| XM_030996837.1 | 20.910 | 0.0001 | 0.004 | LOC578453 | glycine dehydrogenase (decarboxylating), mitochondrial, transcript variant X2 |
| XR_004066239.1 | 20.686 | 0.0001 | 0.005 | LOC575346 | uncharacterized LOC575346 |
| XM_789588.5 | 20.612 | 0.0001 | 0.005 | LOC589961 | 40S ribosomal protein S25 |
| XM_785828.5 | 20.467 | 0.0001 | 0.005 | LOC586030 | cytochrome b-c1 complex subunit 7 |
| XM_030979044.1 | 20.354 | 0.0001 | 0.005 | LOC592738 | 40S ribosomal protein S24 |
| XM_030975807.1 | 20.224 | 0.0002 | 0.005 | LOC105442240 | dynein light chain roadblock-type 2-like |
| XM_030976663.1 | 20.132 | 0.0002 | 0.005 | LOC115920596 | uncharacterized LOC115920596 |
| XR_143788.3 | 19.799 | 0.0002 | 0.006 | LOC753066 | developmentally regulated ectodermal protein |
| XM_788739.4 | 19.743 | 0.0002 | 0.006 | LOC589085 | 60S ribosomal protein L31 |
| XR_004064267.1 | 19.449 | 0.0002 | 0.007 | LOC115924291 | uncharacterized LOC115924291 |
| XM_775045.5 | 19.312 | 0.0002 | 0.007 | LOC574677 | mesoderm induction early response protein 1, transcript variant X2 |
| XM_030991716.1 | 18.897 | 0.0003 | 0.009 | LOC752419 | aqualysin-1 |
| XM_776937.5 | 18.644 | 0.0003 | 0.010 | LOC576654 | 40S ribosomal protein S26, transcript variant X2 |
| XM_030991493.1 | 18.198 | 0.0004 | 0.012 | LOC115926617 | NADH dehydrogenase [ubiquinone] 1 alpha subcomplex subunit 3-like |
| XM_779323.5 | 18.093 | 0.0004 | 0.012 | LOC579195 | protein RER1 |
| XR_004063272.1 | 18.043 | 0.0004 | 0.012 | LOC115922013 | 5.8S ribosomal RNA |
| XM_030998719.1 | 18.069 | 0.0004 | 0.012 | LOC593365 | circularly permutated Ras protein 1, transcript variant X3 |
| XM_030990638.1 | 18.132 | 0.0004 | 0.012 | LOC115926125 | uncharacterized LOC115926125 |
| XM_030989778.1 | 17.649 | 0.0005 | 0.014 | LOC592500 | sulfotransferase |
| XM_777033.5 | 17.718 | 0.0005 | 0.014 | LOC576760 | 60S ribosomal protein L32 |
| XR_004065545.1 | 17.652 | 0.0005 | 0.014 | LOC115927286 | uncharacterized LOC115927286, transcript variant X1 |
| XR_970617.2 | 17.708 | 0.0005 | 0.014 | LOC105437465 | uncharacterized LOC105437465 |
| XM_030991567.1 | 17.311 | 0.0006 | 0.016 | LOC105438529 | small nuclear ribonucleoprotein Sm D3 |
| XM_030973881.1 | 17.227 | 0.0006 | 0.016 | LOC754976 | protein transport protein Sec61 subunit beta |
| NM_214576.3 | 17.228 | 0.0006 | 0.016 | GeneID_373378 | metallothionein b |
| XM_781033.4 | 17.118 | 0.0007 | 0.017 | LOC581007 | heme-binding protein 2 |
| XM_030999610.1 | 17.032 | 0.0007 | 0.017 | LOC579019 | ubiquitin-40S ribosomal protein S27a |
| XR_972543.2 | 16.918 | 0.0007 | 0.018 | LOC105442870 | uncharacterized LOC105442870 |
| XM_001177297.3 | 16.822 | 0.0008 | 0.018 | LOC752200 | phospholipase A2 |
| XM_030987101.1 | 16.756 | 0.0008 | 0.019 | LOC754717 | inactive C-alpha-formylglycine-generating enzyme 2 |
| XM_790000.4 | 16.538 | 0.0009 | 0.020 | LOC590393 | cytochrome b-c1 complex subunit 6, mitochondrial |
| XM_011675147.2 | 16.328 | 0.001 | 0.022 | GeneID_373469 | ribosomal protein S15a |
| XM_030972964.1 | 16.257 | 0.001 | 0.023 | LOC115919367 | 60S ribosomal protein L23 |
| XM_011664497.2 | 16.077 | 0.001 | 0.024 | GeneID_574895 | ribosomal protein S14 |
| XM_791360.5 | 16.003 | 0.001 | 0.025 | LOC591809 | 60S ribosomal protein L35 |
| XM_030984863.1 | 15.974 | 0.001 | 0.025 | LOC586618 | WW domain-binding protein 11 |
| XM_011680115.2 | 15.885 | 0.001 | 0.026 | LOC581803 | 60S ribosomal protein L11 |
| XM_030992750.1 | 15.832 | 0.001 | 0.026 | LOC576067 | 60S ribosomal protein L12 |
| XR_004064036.1 | 15.625 | 0.001 | 0.028 | LOC115923665 | uncharacterized LOC115923665 |
| XM_788832.5 | 15.535 | 0.001 | 0.029 | LOC589183 | 60S acidic ribosomal protein P1 |
| XM_030984544.1 | 15.274 | 0.002 | 0.033 | LOC105445861 | ATP synthase subunit f, mitochondrial-like |
| XM_793298.5 | 15.190 | 0.002 | 0.034 | LOC593840 | NADH dehydrogenase [ubiquinone] 1 subunit C2 |
| XM_774944.5 | 15.158 | 0.002 | 0.034 | LOC577040 | 40S ribosomal protein S16 |
| XM_030999040.1 | 15.094 | 0.002 | 0.034 | LOC115929578 | RAC serine/threonine-protein kinase-like |
| XR_004064409.1 | 14.798 | 0.002 | 0.039 | LOC115924641 | uncharacterized LOC115924641 |
| XR_004063563.1 | 14.440 | 0.002 | 0.046 | LOC115922689 | uncharacterized LOC115922689 |
| XR_004065897.1 | 14.259 | 0.003 | 0.049 | LOC115928120 | uncharacterized LOC115928120 |

***Supplemental Table 3:*** *81 overrepresented GO term annotations for the significantly differentially expressed genes at the gastrula stage. For GO class there are three categories: Molecular Function (MF), Biological Processes (BP) and Cellular Component (CC). These GO terms were associated with organelle development and metabolic processes. Columns highlight the number of annotations, significant annotations and the expected p-value compared to the actual p-value.*

| GO.ID | GO_class | Term | Annotated | Significant | Expected | classic |
| --- | --- | --- | --- | --- | --- | --- |
| GO:0003735 | MF | structural constituent of ribosome | 161 | 31 | 1.61 | < 1e-30 |
| GO:0003723 | MF | RNA binding | 823 | 19 | 8.24 | 0.0005 |
| GO:0031593 | MF | polyubiquitin modification-dependent protein binding | 23 | 3 | 0.23 | 0.002 |
| GO:0140030 | MF | modification-dependent protein binding | 47 | 3 | 0.47 | 0.012 |
| GO:0003729 | MF | mRNA binding | 151 | 5 | 1.51 | 0.018 |
| GO:0019887 | MF | protein kinase regulator activity | 56 | 3 | 0.56 | 0.018 |
| GO:0030234 | MF | enzyme regulator activity | 408 | 9 | 4.09 | 0.021 |
| GO:0015078 | MF | proton transmembrane transporter activity | 61 | 3 | 0.61 | 0.023 |
| GO:0019207 | MF | kinase regulator activity | 62 | 3 | 0.62 | 0.024 |
| GO:0098772 | MF | molecular function regulator activity | 495 | 10 | 4.96 | 0.026 |
| GO:0003676 | MF | nucleic acid binding | 2137 | 29 | 21.4 | 0.046 |
| GO:0005783 | CC | endoplasmic reticulum | 476 | 15 | 5.45 | 0.0003 |
| GO:0005743 | CC | mitochondrial inner membrane | 156 | 8 | 1.79 | 0.0004 |
| GO:0098803 | CC | respiratory chain complex | 56 | 5 | 0.64 | 0.0004 |
| GO:0070469 | CC | respirasome | 57 | 5 | 0.65 | 0.0005 |
| GO:0098796 | CC | membrane protein complex | 440 | 14 | 5.04 | 0.0005 |
| GO:0019866 | CC | organelle inner membrane | 167 | 8 | 1.91 | 0.0006 |
| GO:0031090 | CC | organelle membrane | 937 | 22 | 10.72 | 0.0008 |
| GO:0098800 | CC | inner mitochondrial membrane protein complex | 68 | 5 | 0.78 | 0.001 |
| GO:0031966 | CC | mitochondrial membrane | 206 | 8 | 2.36 | 0.003 |
| GO:0005740 | CC | mitochondrial envelope | 225 | 8 | 2.57 | 0.004 |
| GO:1902554 | CC | serine/threonine protein kinase complex | 47 | 3 | 0.54 | 0.016 |
| GO:0098798 | CC | mitochondrial protein-containing complex | 130 | 5 | 1.49 | 0.017 |
| GO:0005789 | CC | endoplasmic reticulum membrane | 288 | 8 | 3.3 | 0.017 |
| GO:0031967 | CC | organelle envelope | 291 | 8 | 3.33 | 0.018 |
| GO:0031975 | CC | envelope | 291 | 8 | 3.33 | 0.018 |
| GO:0042175 | CC | nuclear outer membrane-endoplasmic reticulum membrane network | 291 | 8 | 3.33 | 0.018 |
| GO:0098827 | CC | endoplasmic reticulum subcompartment | 292 | 8 | 3.34 | 0.019 |
| GO:1902911 | CC | protein kinase complex | 52 | 3 | 0.6 | 0.022 |
| GO:1902494 | CC | catalytic complex | 858 | 16 | 9.82 | 0.035 |
| GO:1990204 | CC | oxidoreductase complex | 68 | 3 | 0.78 | 0.043 |
| GO:0009060 | BP | aerobic respiration | 67 | 6 | 0.77 | 0.0001 |
| GO:0022904 | BP | respiratory electron transport chain | 45 | 5 | 0.52 | 0.0002 |
| GO:0045333 | BP | cellular respiration | 75 | 6 | 0.86 | 0.0002 |
| GO:0032507 | BP | maintenance of protein location in cell | 11 | 3 | 0.13 | 0.0002 |
| GO:0045185 | BP | maintenance of protein location | 11 | 3 | 0.13 | 0.0002 |
| GO:0022900 | BP | electron transport chain | 50 | 5 | 0.57 | 0.0003 |
| GO:0044249 | BP | cellular biosynthetic process | 2876 | 50 | 33.05 | 0.0003 |
| GO:0034641 | BP | cellular nitrogen compound metabolic process | 2696 | 47 | 30.99 | 0.0006 |
| GO:0015980 | BP | energy derivation by oxidation of organic compounds | 91 | 6 | 1.05 | 0.0006 |
| GO:0044237 | BP | cellular metabolic process | 4397 | 67 | 50.53 | 0.0007 |
| GO:0042274 | BP | ribosomal small subunit biogenesis | 62 | 5 | 0.71 | 0.0007 |
| GO:1901576 | BP | organic substance biosynthetic process | 2963 | 50 | 34.05 | 0.0007 |
| GO:0042254 | BP | ribosome biogenesis | 217 | 9 | 2.49 | 0.0009 |
| GO:0009058 | BP | biosynthetic process | 2994 | 50 | 34.41 | 0.001 |
| GO:0006613 | BP | cotranslational protein targeting to membrane | 18 | 3 | 0.21 | 0.001 |
| GO:0006614 | BP | SRP-dependent cotranslational protein targeting to membrane | 18 | 3 | 0.21 | 0.001 |
| GO:0045859 | BP | regulation of protein kinase activity | 21 | 3 | 0.24 | 0.002 |
| GO:0022613 | BP | ribonucleoprotein complex biogenesis | 292 | 10 | 3.36 | 0.002 |
| GO:0006414 | BP | translational elongation | 23 | 3 | 0.26 | 0.002 |
| GO:0043549 | BP | regulation of kinase activity | 23 | 3 | 0.26 | 0.002 |
| GO:0051651 | BP | maintenance of location in cell | 23 | 3 | 0.26 | 0.002 |
| GO:0009987 | BP | cellular process | 8030 | 100 | 92.29 | 0.002 |
| GO:0006091 | BP | generation of precursor metabolites and energy | 121 | 6 | 1.39 | 0.003 |
| GO:0045047 | BP | protein targeting to ER | 26 | 3 | 0.3 | 0.003 |
| GO:0051235 | BP | maintenance of location | 26 | 3 | 0.3 | 0.003 |
| GO:0072599 | BP | establishment of protein localization to endoplasmic reticulum | 26 | 3 | 0.3 | 0.003 |
| GO:0000462 | BP | maturation of SSU-rRNA from tricistronic rRNA transcript (SSU-rRNA, 5.8S rRNA, LSU-rRNA) | 27 | 3 | 0.31 | 0.004 |
| GO:0051338 | BP | regulation of transferase activity | 30 | 3 | 0.34 | 0.005 |
| GO:0031399 | BP | regulation of protein modification process | 62 | 4 | 0.71 | 0.006 |
| GO:0006612 | BP | protein targeting to membrane | 32 | 3 | 0.37 | 0.006 |
| GO:0001932 | BP | regulation of protein phosphorylation | 35 | 3 | 0.4 | 0.007 |
| GO:0042255 | BP | ribosome assembly | 36 | 3 | 0.41 | 0.008 |
| GO:0042325 | BP | regulation of phosphorylation | 38 | 3 | 0.44 | 0.009 |
| GO:0030490 | BP | maturation of SSU-rRNA | 39 | 3 | 0.45 | 0.010 |
| GO:0043170 | BP | macromolecule metabolic process | 4099 | 59 | 47.11 | 0.012 |
| GO:0033365 | BP | protein localization to organelle | 174 | 6 | 2 | 0.015 |
| GO:0008152 | BP | metabolic process | 5346 | 72 | 61.44 | 0.019 |
| GO:0051246 | BP | regulation of protein metabolic process | 189 | 6 | 2.17 | 0.022 |
| GO:0006807 | BP | nitrogen compound metabolic process | 4496 | 62 | 51.67 | 0.029 |
| GO:0044085 | BP | cellular component biogenesis | 832 | 16 | 9.56 | 0.033 |
| GO:0090150 | BP | establishment of protein localization to membrane | 57 | 3 | 0.66 | 0.034 |
| GO:0019220 | BP | regulation of phosphate metabolic process | 58 | 3 | 0.67 | 0.034 |
| GO:0051174 | BP | regulation of phosphorus metabolic process | 58 | 3 | 0.67 | 0.034 |
| GO:0140694 | BP | non-membrane-bounded organelle assembly | 103 | 4 | 1.18 | 0.039 |
| GO:0042273 | BP | ribosomal large subunit biogenesis | 60 | 3 | 0.69 | 0.046 |
| GO:0022618 | BP | protein-RNA complex assembly | 107 | 4 | 1.23 | 0.049 |
| GO:0051668 | BP | localization within membrane | 112 | 4 | 1.29 | 0.002 |
| GO:0071826 | BP | protein-RNA complex organization | 112 | 4 | 1.29 | 0.012 |
| GO:0071840 | BP | cellular component organization or biogenesis | 1923 | 30 | 22.1 | 0.018 |
| GO:0071705 | BP | nitrogen compound transport | 549 | 11 | 6.31 | 0.018 |

***Supplemental Table 4:*** *Significantly differentially expressed genes (57) identified with a likelihood ratio test (LRT) via DeSeq2 (Love et al., 2014) between the treatments at the pluteus stage. Columns show transcript name, the statistical comparison of expression between treatments (stat), the p-value, p-adjust value, gene ID and description. P-values highlight the differences between the full model with mate pair and treatment compared to the reduced model with only mate pair. Using gene ontology to identify gene function, the topmost differentially expressed gene had to do with ribosomal proteins. Other significantly differentially expressed genes were linked to additional ribosomal proteins, tubulin associated genes, zinc finger protein and transcript variants.*

| Transcript | stat | pvalue | padj | ensembl_gene_id | description |
| --- | --- | --- | --- | --- | --- |
| XM_030980614.1 | 52.236 | 2.67E-11 | 1.32E-08 | LOC115922215 | 60S ribosomal protein L31-like |
| XM_030990222.1 | 45.306 | 7.97E-10 | 1.96E-07 | LOC115926013 | metallothionein-A-like |
| XM_030983063.1 | 32.908 | 3.37E-07 | 5.14E-05 | LOC115923028 | 40S ribosomal protein S7-like |
| XM_789831.5 | 32.467 | 4.17E-07 | 5.14E-05 | LOC105436394 | 40S ribosomal protein S18 |
| XM_011682841.2 | 28.319 | 3.11E-06 | 0.0002 | LOC100892690 | galectin-3-binding protein |
| XM_030984515.1 | 28.418 | 2.97E-06 | 0.0002 | LOC373246 | 60S ribosomal protein L18 |
| XM_791138.5 | 28.323 | 3.11E-06 | 0.0002 | LOC591582 | probable 60S ribosomal protein L37-A, transcript variant X1 |
| XM_030995181.1 | 23.194 | 3.68E-05 | 0.002 | LOC115918936 | 40S ribosomal protein S11-like, transcript variant X1 |
| XM_781556.5 | 22.876 | 4.29E-05 | 0.002 | LOC581567 | uncharacterized protein C1orf194 homolog |
| XM_011662433.2 | 21.143 | 9.83E-05 | 0.005 | LOC105436651 | 60S ribosomal protein L14-like, transcript variant X1 |
| XM_777072.5 | 20.991 | 0.0001 | 0.005 | LOC576803 | 60S ribosomal protein L22 |
| XM_030990934.1 | 20.105 | 0.0002 | 0.007 | LOC115926236 | zinc finger protein 593-like |
| XM_030973879.1 | 19.782 | 0.0002 | 0.007 | LOC577852 | 60S ribosomal protein L30, transcript variant X2 |
| XM_030980960.1 | 18.926 | 0.0003 | 0.009 | LOC594617 | nucleoside diphosphate kinase B |
| XM_792139.5 | 18.926 | 0.0003 | 0.009 | LOC592626 | 60S ribosomal protein L34-B |
| XM_030981950.1 | 18.598 | 0.0003 | 0.010 | LOC584812 | 40S ribosomal protein S7 |
| XM_030995657.1 | 17.626 | 0.0005 | 0.015 | LOC105439767 | tubulin alpha-1 chain |
| XM_790998.5 | 17.342 | 0.0006 | 0.017 | LOC591437 | calmodulin-beta |
| XR_972047.2 | 17.196 | 0.0006 | 0.017 | LOC105441516 | neurotrypsin, transcript variant X1 |
| XM_030991923.1 | 16.670 | 0.0008 | 0.020 | LOC588157 | 60S ribosomal protein L23a |
| XM_030984838.1 | 16.318 | 0.001 | 0.023 | LOC115923673 | tubulin beta chain |
| XM_030994829.1 | 16.246 | 0.001 | 0.0226 | LOC115918925 | tubulin alpha-1 chain-like |
| XM_011682867.2 | 16.126 | 0.001 | 0.023 | LOC577036 | tubulin beta chain |
| XM_030994609.1 | 15.784 | 0.001 | 0.026 | LOC105445674 | 60S ribosomal protein L13a-like |
| XM_031000341.1 | 15.614 | 0.001 | 0.027 | LOC115930019 | meiosis expressed gene 1 protein homolog |
| XM_030979044.1 | 15.322 | 0.002 | 0.030 | LOC592738 | 40S ribosomal protein S24 |
| XM_030994862.1 | 15.190 | 0.002 | 0.030 | LOC587551 | 60S ribosomal protein L24 |
| XM_788739.4 | 14.963 | 0.002 | 0.031 | LOC589085 | 60S ribosomal protein L31 |
| XM_793320.5 | 15.037 | 0.002 | 0.031 | LOC593861 | 60S ribosomal protein L19 |
| XR_004064978.1 | 14.825 | 0.002 | 0.032 | LOC105441516 | neurotrypsin, transcript variant X1 |
| XM_778057.5 | 14.564 | 0.002 | 0.036 | LOC577852 | 60S ribosomal protein L30, transcript variant X2 |
| XR_971323.2 | 14.347 | 0.003 | 0.0002 | LOC105439540 | uncharacterized LOC105439540 |
| XM_001200305.4 | 13.800 | 0.003 | 0.0002 | LOC764101 | protein LLP homolog |
| XM_011671068.2 | 13.793 | 0.003 | 0.0002 | LOC100893456 | elongation of very long chain fatty acids protein 6 |
| XM_030983020.1 | 13.518 | 0.004 | 0.002 | LOC105441189 | 40S ribosomal protein S21 |
| XM_776941.5 | 13.509 | 0.004 | 0.003 | LOC576658 | 40S ribosomal protein S20 |
| XM_779989.5 | 13.464 | 0.004 | 0.005 | LOC579897 | 60S ribosomal protein L35a |

***Supplemental Table V:*** *34 overrepresented GO term annotations for the significantly differentially expressed genes at the pluteus stage. For GO class there are three categories: Molecular Function (MF), Biological Processes (BP) and Cellular Component (CC). These GO terms were associated with genes involved in microtubule processes and skeletal development. Columns highlight the number of annotations, significant annotations and the expected p-value compared to the actual p-value.*

| GO.ID | GO_class | Term | Annotated | Significant | Expected | classic |
| --- | --- | --- | --- | --- | --- | --- |
| GO:0003723 | MF | RNA binding | 765 | 9 | 3.08 | 0.003 |
| GO:0005525 | MF | GTP binding | 274 | 5 | 1.1 | 0.005 |
| GO:0019001 | MF | guanyl nucleotide binding | 277 | 5 | 1.12 | 0.005 |
| GO:0032561 | MF | guanyl ribonucleotide binding | 277 | 5 | 1.12 | 0.005 |
| GO:0097159 | MF | organic cyclic compound binding | 3392 | 20 | 13.66 | 0.026 |
| GO:0003676 | MF | nucleic acid binding | 1901 | 13 | 7.65 | 0.030 |
| GO:0099513 | CC | polymeric cytoskeletal fiber | 198 | 6 | 0.83 | 0.0002 |
| GO:0099081 | CC | supramolecular polymer | 219 | 6 | 0.91 | 0.0003 |
| GO:0099512 | CC | supramolecular fiber | 219 | 6 | 0.91 | 0.0003 |
| GO:0015935 | CC | small ribosomal subunit | 53 | 3 | 0.22 | 0.001 |
| GO:0099080 | CC | supramolecular complex | 316 | 6 | 1.32 | 0.002 |
| GO:0043226 | CC | organelle | 5135 | 30 | 21.45 | 0.003 |
| GO:0032991 | CC | protein-containing complex | 2148 | 17 | 8.97 | 0.003 |
| GO:0015630 | CC | microtubule cytoskeleton | 373 | 6 | 1.56 | 0.004 |
| GO:0043229 | CC | intracellular organelle | 5042 | 28 | 21.07 | 0.014 |
| GO:0005737 | CC | cytoplasm | 3988 | 23 | 16.66 | 0.027 |
| GO:0042254 | BP | ribosome biogenesis | 218 | 6 | 0.84 | 0.0002 |
| GO:0000226 | BP | microtubule cytoskeleton organization | 224 | 6 | 0.86 | 0.0002 |
| GO:0022613 | BP | ribonucleoprotein complex biogenesis | 291 | 6 | 1.12 | 0.0007 |
| GO:0071840 | BP | cellular component organization or biogenesis | 1765 | 15 | 6.8 | 0.001 |
| GO:0010467 | BP | gene expression | 2008 | 16 | 7.74 | 0.001 |
| GO:0019538 | BP | protein metabolic process | 1809 | 15 | 6.97 | 0.001 |
| GO:1901564 | BP | organonitrogen compound metabolic process | 2273 | 17 | 8.76 | 0.002 |
| GO:0007017 | BP | microtubule-based process | 373 | 6 | 1.44 | 0.003 |
| GO:0044249 | BP | cellular biosynthetic process | 2621 | 18 | 10.1 | 0.003 |
| GO:0007010 | BP | cytoskeleton organization | 409 | 6 | 1.58 | 0.004 |
| GO:1901576 | BP | organic substance biosynthetic process | 2701 | 18 | 10.41 | 0.004 |
| GO:0009059 | BP | macromolecule biosynthetic process | 2260 | 16 | 8.71 | 0.005 |
| GO:0009058 | BP | biosynthetic process | 2734 | 18 | 10.54 | 0.005 |
| GO:0000278 | BP | mitotic cell cycle | 198 | 4 | 0.76 | 0.007 |
| GO:0007049 | BP | cell cycle | 333 | 5 | 1.28 | 0.008 |
| GO:0044085 | BP | cellular component biogenesis | 788 | 8 | 3.04 | 0.008 |
| GO:0006996 | BP | organelle organization | 984 | 9 | 3.79 | 0.010 |
| GO:0034641 | BP | cellular nitrogen compound metabolic process | 2490 | 16 | 9.6 | 0.013 |
